# Urea as a By-Product of Ammonia Metabolism Can Be a Potential Serum Biomarker of Hepatocellular Carcinoma

**DOI:** 10.3389/fcell.2021.650748

**Published:** 2021-04-01

**Authors:** Changsen Bai, Hailong Wang, Dong Dong, Tong Li, Zhi Yu, Junfei Guo, Wei Zhou, Ding Li, Ruochen Yan, Liyan Wang, Zhaosong Wang, Yueguo Li, Li Ren

**Affiliations:** ^1^Department of Laboratory, Tianjin Medical University Cancer Institute and Hospital, National Clinical Research Center for Cancer, Key Laboratory of Cancer Prevention and Therapy, Tianjin’s Clinical Research Center for Cancer, Tianjin, China; ^2^Department of Cancer Cell Biology, Tianjin Medical University Cancer Institute and Hospital, National Clinical Research Center for Cancer, Key Laboratory of Cancer Prevention and Therapy, Tianjin’s Clinical Research Center for Cancer, Tianjin, China; ^3^Department of Laboratory, Second Affiliated Hospital of Tianjin University of TCM, Tianjin, China; ^4^Department of Laboratory, First Affiliated Hospital of Shaoyang University, Shaoyang, China; ^5^Department of Laboratory, Guangdong Women and Children Hospital, Guangzhou, China; ^6^School of Medical Laboratory, Tianjin Medical University, Tianjin, China; ^7^Department of Public Laboratory, Tianjin Medical University Cancer Institute and Hospital, National Clinical Research Center for Cancer, Key Laboratory of Cancer, Prevention and Therapy, Tianjin’s Clinical Research Center for Cancer, Tianjin, China

**Keywords:** metabolomics, hepatocellular carcinoma, urea, ammonia, CPS1, biomarker

## Abstract

Hepatocellular carcinoma (HCC) is highly malignant; nearly half of the new cases and deaths are in China. The poor prognosis of HCC is mainly due to late diagnosis; many new biomarkers have been developed for HCC diagnosis. However, few markers are quickly translated into clinical practice; early and differential diagnosis of HCC from cirrhosis and/or hepatitis is still a clinical challenge. Metabolomics and biochemical methods were used to reveal specific serum biomarkers of HCC. Most of the elevated metabolites in HCC and HBV patients were overlapped compared with controls. Urea was the specifically elevated serum biomarker of HCC patients. Moreover, urea combined with AFP and CEA can improve the sensitivity of HCC diagnosis. The plasma ammonia of HCC patients was significantly higher than healthy controls. Co-culture cell model revealed normal liver cells cooperated with cancer cells to metabolize ammonia into urea. The urea metabolism in cancer cells marginally depended on the expression of CPS1. However, the expression of CPS1 did not change with ammonium chloride, which might regulate the urea cycle through enzyme activity. The urea cycle could detoxify high concentrations of ammonia to promote cancer cell proliferation. Therefore, urea was a by-product of ammonia metabolism and could be a potential serum biomarker for HCC. The combined application of metabolomics and biochemical methods can discover new biomarkers for the early diagnosis of HCC and be quickly applied to clinical diagnosis.

## Introduction

According to annual forecasts, the World Health Organization estimates that more than 1 million patients will die of liver cancer by 2030. New cases and deaths related to liver cancer in China account for about 50% of the world (Torre et al., 2015; Fu and Wang, 2018). Hepatocellular carcinoma (HCC) is highly malignant and usually diagnosed at an advanced stage, accounting for over 80% of primary liver cancers (Forner et al., 2018). Early diagnosis and a better understanding of the molecular mechanisms leading to HCC occurrence and progression are clinically urgent. At present, despite tremendous efforts have been made to discover new biomarkers for early diagnosis of HCC, the diagnosis of HCC still depends on imaging (ultrasound B, CT or MRI) and alpha-fetoprotein (AFP) in clinical practice (Montal et al., 2019; Timo Alexander et al., 2020; Zech et al., 2020). However, their sensitivity and/or specificity are not satisfactory.

Metabolic reprogramming is a recognized hallmark of cancer (Hanahan and Weinberg, 2011). Metabolites can regulate gene and protein expressions, and metabolic proteins and/or metabolites are potential diagnostic and prognostic biomarkers (DeBerardinis and Chandel, 2016; Pavlova and Thompson, 2016; Wolpaw and Chi, 2018). Reliable results have been obtained using certain metabolites, such as lactate and amino acid, and their changes in serum reflect the metabolic changes in tumor tissue (De Matteis et al., 2018; Fujiwara et al., 2018). Many studies were devoted to discovering serum biomarkers of HCC diagnosis from the aspect of metabolism. Ping Luo et al. (Luo and Yin, 2018) defined a group of biomarkers of serum metabolites, including phenylalanyl-tryptophan and glycine cholate. This panel has a higher diagnostic performance than AFP in distinguishing HCC from high-risk cirrhosis populations, with the area under the receiver-operating characteristic curve (ROC) of 0.807 for the panel versus 0.650 for AFP in the validation set. Tomoyoshi Soga et al. (2011) revealed that c-glutamyl dipeptides are new biomarkers for liver disease and can distinguish different liver disease forms. These studies may reflect various metabolic aspects of HCC. Still, the limited study cohort or lack of sufficient validation restricts further clinic applications of these biomarkers, because most laboratories do not have mass spectrometers for detecting metabolites, especially in developing countries. Moreover, they failed to elucidate the underlying molecular mechanism.

Second to glucose, cancer cells are highly dependent on glutamine for survival and proliferation. Glutamine catabolism is accompanied by the secretion of alanine and ammonia, leading to most of the amino groups of glutamine are lost from the cell instead of being incorporated into other molecules (DeBerardinis et al., 2007). Together with other amino acid metabolism, they lead to the accumulation of ammonia in the tumor microenvironment. Interestingly, ammonia metabolism plays different roles in cancers. Most researchers believe ammonia is a toxic cellular by-product of glutamine metabolism (Khan et al., 2019; Li et al., 2019) and needs to be metabolized into a non-toxic form, such as urea, to be excluded from the body. But Jessica B et al. prove that breast cancer cells can recycle glutamine amide to support biosynthesis (Spinelli and Yoon, 2017). Therefore, the role of ammonia in cancer cells remains to be determined.

The liver represents a perfect metabolic model that governs body energy metabolism through different metabolites’ physiological regulation, including sugars, lipids, amino acids, and the urea cycle. Therefore, our research uses quantitative targeted metabolomics to screen for specific metabolites of HCC and in-depth verification using biochemical analysis methods. We found that urea was a potential biomarker of HCC, and combining it with AFP and CEA could improve the detection efficiency of HCC. Normal liver cells and cancer cells cooperated to metabolize ammonia into urea, leading to the elevated serum urea in HCC patients. The urea cycle could detoxify high concentration of ammonia to promote cancer cell proliferation.

## Materials and Methods

### Study Design and Participants

In this study, we collected 10 serum samples of healthy controls, 10 HBV without cirrhosis and 10 HBV positive HCC patients evolved from cirrhosis with similar sex and age. Hence, the data was comparable between both groups. The metabolites of the serum were analyzed by quantitative targeted metabolomics for differential analysis to find specific potential markers of HCC patients. The biochemical analysis method was used for in-depth analysis and verification. The serum of 115 cases of healthy controls, 69 cases of HBV, 108 cases of HBV positive cirrhosis, 95 cases of HCV, 294 cases of HBV positive HCC patients, and 77 cases of metastatic HCC patients (other cancers metastasize to the liver) were preliminary verified as the training set. Next, the serum of 118 normal healthy controls, 142 cases of lung cancer patients, 150 cases of breast cancer patients, 140 cases of colorectal cancer patients, and 165 cases of HBV positive HCC patients were analyzed as the validation set. All tumor patients were at the early stage, without treatment and metastasis to other sites (except for metastatic HCC). The groups’ age and gender in the training set and validation set were similar, except for the breast cancer group (Supplementary Tables 1, 2). All serum samples were collected on the first day after hospitalization, before surgery or treatment, and quick-freeze at −80°C until use. The exclusion criteria for healthy controls were abnormal liver and kidney function, with a history of liver disease or other systemic diseases. All tumor types were diagnosed by pathology. This study was approved by the Ethics Committee of Tianjin Medical University Cancer Hospital, and all patients signed informed consent.

### Targeted Metabolomics

The frozen serum samples were thawed at 4°C. Take 100 μL of serum to a 1.5 mL Ep tube and add 4 volumes of extract made of MeOH/Acetonitrile (ACN) = (1/1, v/v), containing 0.05 mM DL-Methionine sulfone as an internal standard. After shaking and mixing well, place the protein precipitate at −20°C for 2 h. Centrifuge at 20,000 *g* at 4°C for 10 min, and transfer the upper layer to a new 1.5 mL Ep tube. Spin to dry *in vacuo* by Eppendorf Concentrator 5301, add 100 μL ACN/H_2_O (1/1, v/v), and reconstitute by shaking. Filter with a 0.22 μm filter membrane, and transfer the sample to the sample bottle for testing. The sample injection volume was 10 μL/time, separated by Agilent 1260 Infinity HPLC/PRF/5 liquid phase system, and detected by Agilent 6460 QQQ at Aksomics (Shanghai, China). The analysis was performed on an Amide column (3.5 μm, 2.1 ^∗^ 150 mm, Waters) with a column temperature of 40°C. Mobile phase A is an aqueous solution containing 25 mM NH_4_OAc, 25 mM NH_3_H_2_O, and liquid B is an acetonitrile solution. The column is equilibrated with 90% liquid B. The sample is loaded onto the chromatographic column by the auto-sampler and then separated by the chromatographic column at a flow rate of 0.4 mL/min. Mass spectrometry detection mode and metabolite detection condition settings: Multiple reaction monitoring (MRM) mode detections, select the detection polarity corresponding to the specific metabolite, retention time, and optimized ion pair for detection. Metabolite detection data is quantified using QuantAnalysis software. Perform differential analysis on the quantitative results to obtain corresponding differentially expressed metabolites.

### Biochemical Analyses

Serum and plasma samples were stored at −80°C until further analysis. Urea, creatinine (Cr), and uric acid were detected using an automatic biochemical analyzer AU5800 (Beckman Coulter, United States). Urea was detected as previously described (Wang et al., 2019). Briefly, urea is hydrolyzed by urease to produce ammonia and carbon dioxide. Ammonia and a-ketoglutarate are converted to glutamate under the catalysis of GDH. At the same time, a molar equivalent of reduced NADH is oxidized. For each molecule of urea to be hydrolyzed, two NADH molecules are oxidized. Due to the disappearance of NADH, the absorbance at 340 nm is proportional to the urea concentration in the sample. Cr was measured by enzyme colorimetry. Briefly, creatinine is catalyzed by creatininase to generate creatine, and creatine is catalyzed by creatase to generate sarcosine and urea, and then sarcosine is oxidized to generate glycine and hydrogen peroxide; the red intensity of the produced quinoneimine is proportional to the concentration of Cr, and the intensity can be determined by measuring the increase in absorbance. Uric acid was determined by enzymatic colorimetry. Uricase breaks down uric acid to produce allantoin and hydrogen peroxide. In the presence of peroxidase, 4-aminopyrine generates quinoneimine dye from hydrogen peroxide. The red intensity of the produced quinoneimine is proportional to the concentration of uric acid.

Ammonia was detected by VITROS 5600 Integrated System (Ortho Clinical Diagnostics, United States) as previously described (Wang et al., 2019). Briefly, a drop of sample is deposited on the slide and then evenly distributed to the underlying layers. Water and non-protein components travel to the underlying buffered reagent layer, and ammonium ions are converted into gaseous ammonia. The semi-permeable membrane only allows ammonia gas to pass through and prevents buffer or hydroxide ions from reaching the indicator layer. After a period of time, the white background of the extended layer is used as a diffuse reflector to measure the reflection density of the dye.

### Cell Culture

The human HL-7702 hepatocyte cell line and human HCC cell line MHCC97H were obtained from the Liver Cancer Institute, Zhongshan Hospital, Fudan University, Shanghai, China. HepG2, PLC/PRF/5, Hep3B, A549, MCF-7, NCM460, HCT116, HCT8 cells were obtained from ATCC. HLE cell line was from the Health Science Research Resources Bank (Osaka, Japan). All the cells were maintained in high glucose DMEM supplemented with 10% fetal bovine serum (BioInd, Israel) and 50 IU penicillin/streptomycin (Invitrogen, United States) in a humidified atmosphere with 5% CO2 at 37°C. All cell lines were free of mycoplasma contamination and cultured for no more than 2 months.

### Co-Culture Cell Models

200,000 HL-7702 and 100,000 MHCC97H + 100,000 HL-7702 cells were seeded in triplicate in 6-well plates. The next day the medium was changed by fresh DMEM with 10% dialysis fetal bovine serum, 25 mM glucose, and 2 mM glutamine. After 48 h, the medium was collected for urea detection. Falcon embedded cell culture chamber (PET track-etched membrane, 6 well formats, 0.4 μm, Corning, Cat#353090) was used for the co-culture model. 200,000 HL-7702, MHCC97H or PLC/PRF/5 were seeded in triplicate in 6-well plates (lower layer). 100,000 HL-7702 for each cell line were seeded in triplicate in the chamber (upper layer) on new 6-well plates. The next day the medium in 6-well plates and the chamber were changed by fresh DMEM with 10% dialysis fetal bovine serum, 25 mM glucose, and 2 mM glutamine, respectively. Then transfer the chamber to the six-well plate inoculated with HL-7702, MHCC97H or PLC/PRF/5 for culture. After 48 h, medium was collected for urea and ammonia detection.

### Ammonia and Urea Excretion

200,000 cells were seeded in triplicate in 12-well plates. The next day the medium was changed by fresh DMEM with 10% dialysis fetal bovine serum, 25 mM glucose and 2 mM glutamine. After 8 h the medium was collected for ammonia detection. 100,000 cells were seeded in triplicate in 12-well plates. The next day the medium was changed by fresh DMEM with 10% dialysis fetal bovine serum, 25 mM glucose, and 2 mM glutamine. After 48 h, the medium was collected for urea detection. The medium without cells under the same conditions was used to measure the concentration of metabolites as the background control. Values for ammonia and urea in the experimental conditions were subtracted from the respective control media values.

### Proliferation Assays

20,000 cells were seeded in triplicate in 24-well plates, and changed to the conditioned medium the next day. After 3 days, wells were washed twice with PBS buffer to remove dead cells, and then the cells were trypsinized; cell number was determined using a hemocytometer. For each well, the fold change in cell number relative to Day_0_ was calculated.

### Colony Formation Assay

Colony formation assays were performed to assess the effects of NH_4_Cl on cell proliferation. 1000 cells were seeded in triplicate in 12-well plates, change to 2 ml of culture medium containing 0–20 mM of NH_4_Cl the next day. After incubating at 37°C for 2 weeks, cells were stained with 0.5% crystal violet for 5 min, and colonies were counted with Image J.

### Western Blot

After desired treatments as specified as indicated, cells were washed twice with PBS and lysed in buffer on ice (20 mM Tris–HCl, pH 7.5, 150mM NaCl, 1 mM EDTA, 1% Triton X-100, 2.5 mM sodium pyrophosphate, 1mM β-glycer-ophosphate, 1mM sodium vanadate, 1mgml-1 leupeptin, 1mM phenylmethyl-sulfonylfluoride). Equal amounts of protein (30 μg) were loaded onto 10% SDS–PAGE gels. Western detection was carried out using a Li-Cor Odyssey image reader (Li-Cor, United States). The goat anti-rabbit IgG (Cat#C30502-01) and goat anti-mouse IgG (Cat#C30509-01) secondary antibodies were obtained from Li-Cor (United States). The final concentration of the secondary antibodies used was 0.1 μg/ml (1:10000 dilution). The primary antibodies against β-Actin (Cat#60008-1, 1:5000 dilution), GAPDH (Cat#60004-1-Ig, 1:5000 dilution), FLAG Tag (Cat#80010-1-RR, 1:3000 dilution), α-Tubulin (Cat#11224-1-AP, 1:5000 dilution), CPS1 (Cat#18703-1-AP, 1:1000 dilution), OTC (Cat#26470-1-AP, 1:1000 dilution), ARG1 (Cat#16001-1-AP, 1:1000), GDH1 (Cat#14299-1-AP, 1:1000 dilution), and GS (Cat#11037-2-AP, 1:1000 dilution) were purchased from Proteintech (United States), The primary antibodies against CAD (Cat#sc-376072, Santa Cruz, United States) and p-CAD (Ser1859) (Cat#70307, Cell Signaling Technology, United States) were used with a dilution of 1:1000.

### Gene Construction and Lentivirus Production

The pLKO.1 lentiviral RNAi expression system was used to construct lentiviral shRNA for genes. The sequences of shRNA used in this study included the following: shScramble: CCTAAGGTTAAGTCGCCCTCG, shCPS1-#1: CCAGAAATTAAGAACGTCGTA, shCPS1-#2: CGTACTTCAATCAATGTTGTT. pCDH-FLAG-CPS1 plasmid was kindly gifted by Professor Peng Jiang at Tsinghua University. Viral packaging was done according to the protocol: expression plasmids pCDH-CMV-cDNA, pCMV-dR8.91, and pCMV-VSV-G were co-transfected into 293T cells using the Polyethylenimine (PEI) coprecipitation at 20:10:10 μg (for a 10-cm dish). After incubation for 6 h, the medium containing PEI and plasmid mixture was replaced with complete fresh medium. Media containing virus was collected 48 h after transfection and then filter the culture medium with a 0.2 μm filter (Merck Millipore Ltd., IRELAND). The culture medium with the virus was stored at −80°C for use. Cancer cells were infected with the viruses in the presence of polybrene (10 μgml^–1^) for 8 h, the medium was replaced with complete fresh medium for 40 h, and then cells were selected with puromycin, generating stable CPS1 knockdown or over-expression cell lines.

### Statistical Analysis

Quantitative data was expressed as data plots; non-normally distributed data was expressed as median (25% Percentile, 75% Percentile), and normally distributed data was expressed as mean ± SD. Differences between two independent groups were compared with the Mann-Whitney U test. One-way ANOVA followed by Tukey’s multiple comparison tests were used for multi comparisons. The two-tailed value of *P* < 0.05 is statistically significant. GraphPad Prism 8.0 software was adopted to analyze the above data. MedCalc software was used to draw ROC curves of different indicators. The 95% confidence interval for comparing the Area Under Curve (AUC) and *P*-values of related ROC curves were obtained by the method described by DeLong et al. (1988).

## Results

### Screening for Differential Serum Metabolites of HCC by Targeted Metabolomics

**FIGURE 1:**
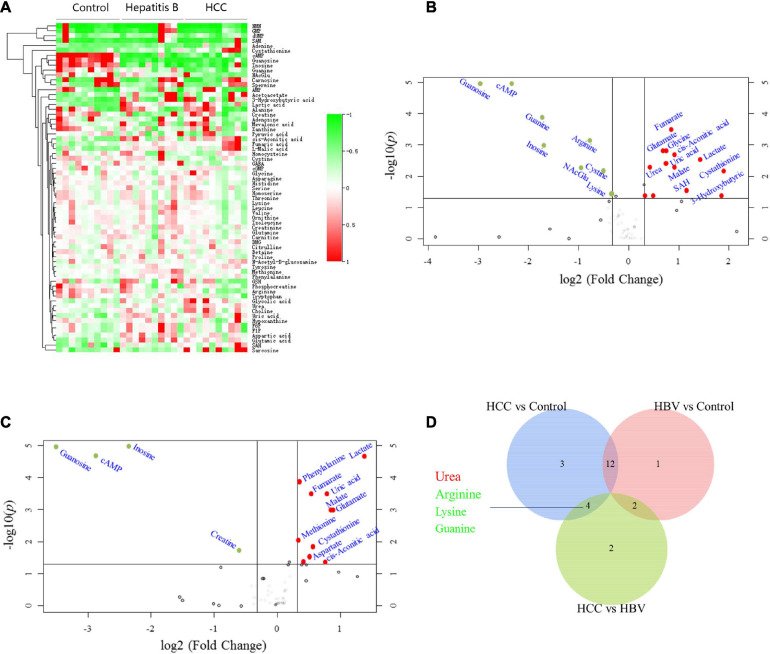
Screening for differential serum metabolites of HCC by targeted metabolomics. **(A)** Heatmap clustering analysis of differential metabolites in serum of 10 normal controls, 10 HBV and 10 HBV positive HCC patients. **(B)** Metabolites volcano map of HCC vs control. Compared with the control group, the significantly increased metabolites were shown in red, and the significantly decreased metabolites were shown in green. **(C)** Metabolites volcano map of HBV vs control. Compared with the control group, the significantly increased metabolites were shown in red, and the significantly decreased metabolites were shown in green. **(D)** Venn diagram was used to characterize different metabolites of HCC vs control, HBV vs control and HCC vs HBV.

To screen for specific serum metabolites of HCC patients can be verified by biochemical methods, we quantitatively detected 135 metabolites, including glycolysis, tricarboxylic acid (TCA) cycle, amino acids, nucleotides, CoA, etc., in the serum of normal people, HBV and HCC patients through targeted metabolomics. Cluster analysis was performed on the 67 different metabolites detected (*P* < 0.05) (Figure 1A). Compared with healthy controls, 11 serum metabolites of HCC significantly increased, including lactate, glycolic acid, cis-Aconitic acid, fumarate, malate, urea, glutamate, aspartate, choline, uric acid, and 3-Hydroxybutyric acid, and 8 decreased, consist of cystine, arginine, lysine, tryptophan, cAMP, guanine, guanosine and inosine (Figure 1B and Table 1). Compared with healthy controls, 11 serum metabolites of HBV significantly increased, including lactate, phenylalanine, fumarate, uric acid, malate, glutamate, methionine, cystathionine, aspartate, glycolic acid, and cis-Aconitic acid, and 4 decreased, consist of guanosine, cAMP, inosine and creatine (Figure 1C and Table 1). To find the specific serum metabolites of HCC patients, we used a Venn diagram to analyze three groups of different metabolites. Most of the elevated metabolites (including lactate, fumarate, malate, and glutamate et al) in HCC and HBV patients were overlapped; urea was the specifically elevated biomarker of HCC patients, arginine, lysine and guanine were the specifically decreased markers of HCC (Figure 1D). MetaboAnalyst was used for KEGG pathway analysis of differential metabolites (Chong et al., 2018). Compared with healthy controls, HCC and HBV patients were both enriched in arginine biosynthesis, alanine, aspartate and glutamate metabolism, D-glutamine and D-glutamate metabolism, pyruvate metabolism, citrate cycle (TCA cycle), cysteine and methionine metabolism, and arginine and proline metabolism pathways (Supplementary Figures 1A,B). Purine metabolism was a differential metabolic pathway in patients with HCC compared with HBV patients (Supplementary Figures 1A,B).

**TABLE 1:** The main differential metabolites of HCC, hepatitis B patients and healthy controls by targeted metabolomics.

Metabolites (g/L)	Control (NC)	Hepatitis B (HBV)	HCC	*P*-value (HCC/NC)	Fold change (HCC/NC)	*P*-value (HBV/NC)	Fold change (HBV/NC)	*P*-value (HCC/HBV)	Fold change (HCC/HBV)
Lactate	4.50 ± 1.38	11.69 ± 6.07	12.13 ± 9.35	0.0200	2.7	0.0018	2.6	0.9034	1.04
Glycolic acid	0.10 ± 0.02	0.13 ± 0.03	0.16 ± 0.07	0.0072	1.68	0.0267	1.31	0.1415	1.28
cis-Aconitic acid	0.05 ± 0.02	0.08 ± 0.04	0.09 ± 0.03	0.0028	1.9	0.0203	1.7	0.5490	1.12
Fumarate	0.01 ± 0.00	0.02 ± 0.00	0.03 ± 0.01	0.0063	1.8	0.0003	1.45	0.1868	1.25
L-Malate	0.69 ± 0.00	1.25 ± 0.12	1.30 ± 0.78	0.0355	1.88	0.0001	1.81	0.8465	1.04
Cystine	1.27 ± 0.24	1.19 ± 0.44	0.90 ± 0.26	0.0035	0.7	0.6096	0.94	0.0837	0.75
Arginine	95.96 ± 21.10	81.52 ± 24.13	56.16 ± 20.74	0.0005	0.59	0.1712	0.85	0.0214	0.69
Urea	25.93 ± 4.27	28.24 ± 5.25	34.61 ± 6.77	0.0030	1.33	0.2966	1.09	0.0303	1.23
GABA	0.12 ± 0.03	0.13 ± 0.04	0.09 ± 0.02	0.0289	0.76	0.7805	1.04	0.0246	0.74
Glutamate	7.53 ± 2.19	13.89 ± 4.23	12.13 ± 2.62	0.0005	1.61	0.0005	1.84	0.2765	0.87
Histidine	374.06 ± 41.81	378.28 ± 70.96	313.19 ± 62.85	0.0201	0.84	0.8731	1.01	0.0435	0.83
Aspartate	1.06 ± 0.16	1.51 ± 0.44	1.33 ± 0.32	0.0282	1.25	0.0074	1.42	0.3075	0.88
Lysine	29.96 ± 6.22	31.80 ± 7.41	23.71 ± 5.68	0.0306	0.79	0.5551	1.06	0.0135	0.75
Tiyptophan	800.96 ± 190.34	691.64 ± 139.92	607.54 ± 205.72	0.0426	0.76	0.1606	0.86	0.2992	0.88
Choline	96.59 ± 17.93	103.30 ± 32.86	135.92 ± 44.61	0.0186	1.41	0.5777	1.07	0.0791	1.32
Sarcosine	0.12 ± 0.10	0.10 ± 0.02	0.23 ± 0.15	0.0729	1.94	0.5657	0.83	0.0152	2.33
cAMP	0.17 ± 0.04	0.02 ± 0.03	0.03 ± 0.02	0.0000	0.2	0.0000	0.14	0.3479	1.46
Guanine	0.09 ± 0.07	0.05 ± 0.02	0.03 ± 0.01	0.0151	0.3	0.0964	0.54	0.0084	0.56
Guanosine	15.76 ± 9.04	1.38 ± 0.74	2.02 ± 1.57	0.0002	0.13	0.0001	0.09	0.2593	1.46
Inosine	115.85 ± 46.88	22.61 ± 21.65	35.81 ± 59.90	0.0037	0.31	0.0000	0.2	0.5206	1.58
Uric acid	0.05 ± 0.02	0.09 ± 0.02	0.09 ± 0.03	0.0054	1.67	0.0001	1.73	0.7918	0.97
3-Hydroxybutyric acid	0.11 ± 0.04	0.27 ± 0.20	0.40 ± 0.40	0.0347	3.61	0.0283	2.41	0.3611	1.5

### Serum Urea Was a Potential Biomarker for HCC

**FIGURE 2:**
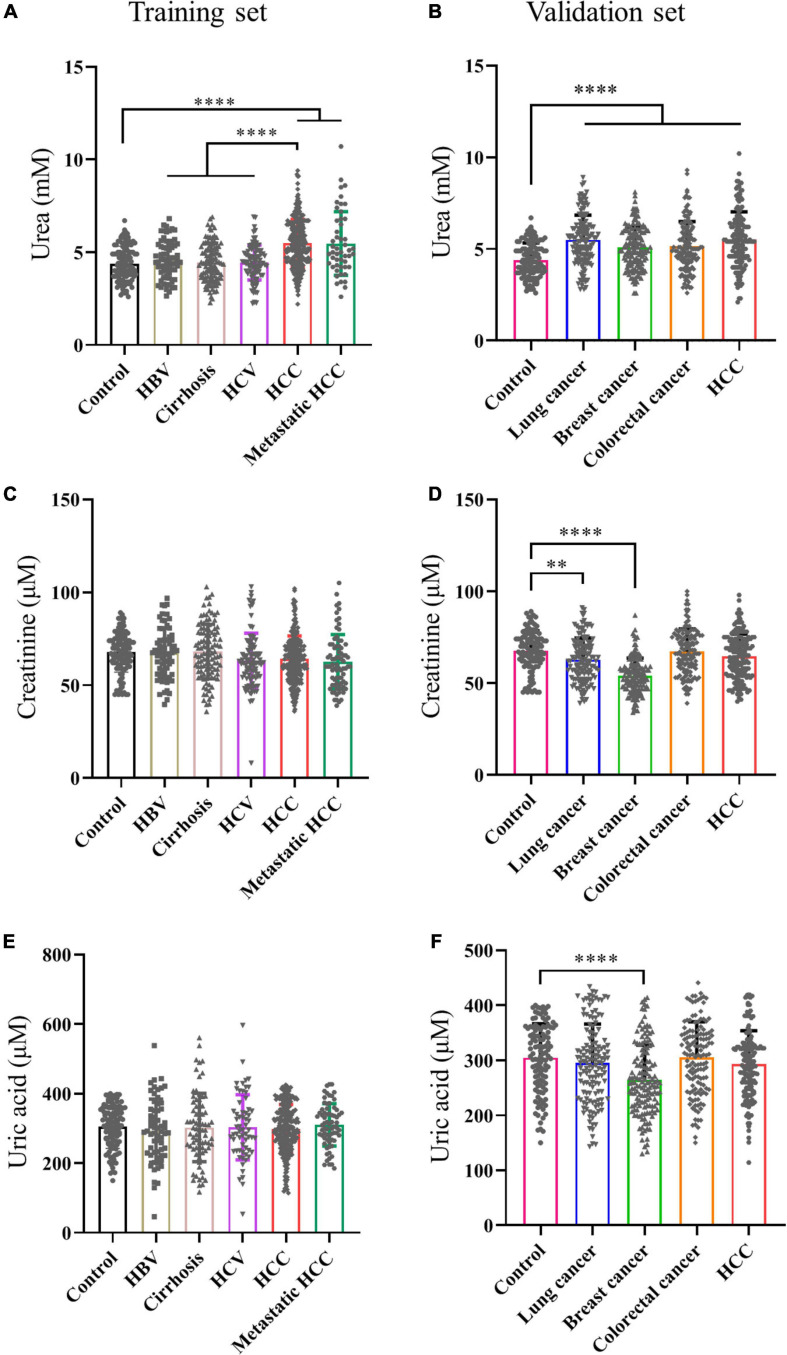
Serum urea was a potential biomarker for HCC. **(A,C,E)** Scatter plots with bar of urea, creatinine and uric acid of control, HBV, Cirrhosis, HCV, HCC and metastatic HCC patients in training set. **(B,D,F)** Scatter plots with bar of urea, creatinine and uric acid of control, lung cancer, breast cancer, colorectal cancer and HCC patients in validation set. ^∗^*p* < 0.05; ^∗∗^*p* < 0.01, ^∗∗∗^*p* < 0.001, ^****^p < 0.0001.

To verify the results of metabolomics, we used biochemical methods to detect metabolites. First of all, to find specific markers in the transition from HBV to HCC, serums of normal people, HBV, HBV positive cirrhosis, HCV, HBV positive HCC, and metastatic HCC patients were collected for verification. Our results showed serum urea in HCC and metastatic HCC patients were significantly higher than healthy controls in the training set (Figure 2A). Moreover, serum urea of HCC was much higher than HBV, Cirrhosis, and HCV patients (Figure 2A), indicating urea can distinguish HCC from other liver diseases. The liver is the metabolic center of the body; many metabolic enzymes are specifically expressed in the liver, we wander to know if other cancers are different from HCC. Therefore, serum samples of patients with HCC, colorectal cancer, lung cancer and breast cancer were collected for multi-tumor analysis. We found serum urea in patients with lung cancer, gastric cancer, colorectal cancer, breast cancer, and HCC were significantly higher than healthy controls in the validation set (Figure 2B), which revealed that elevated serum urea in cancer patients might be a common phenomenon. Next, we asked whether the glomerular filtration and renal tubular reabsorption in cancer patients lead to increased serum urea. We tested creatinine and uric acid to evaluate glomerular filtration and renal tubular reabsorption, respectively. The serum creatinine level in HCC and metastatic HCC patients did not increase significantly compared with healthy controls, HBV, Cirrhosis, and HCV patients (Figure 2C). The serum creatinine of patients with lung cancer and breast cancer was even lower than that of healthy controls (Figure 2D). Metabolomics results showed uric acid in patients with HCC and HBV were significantly higher than those in healthy controls. However, we detected serum uric acid in patients with HCC and various tumors did not increase in the training set and validation set (Figures 2E,F). Taken together, these data demonstrated it was not the abnormal renal function in cancer patients leading to the elevated urea; urea might be a biomarker for HCC.

### Serum Urea Combined With AFP and CEA Can Improve the Diagnostic Efficiency of HCC

**FIGURE 3:**
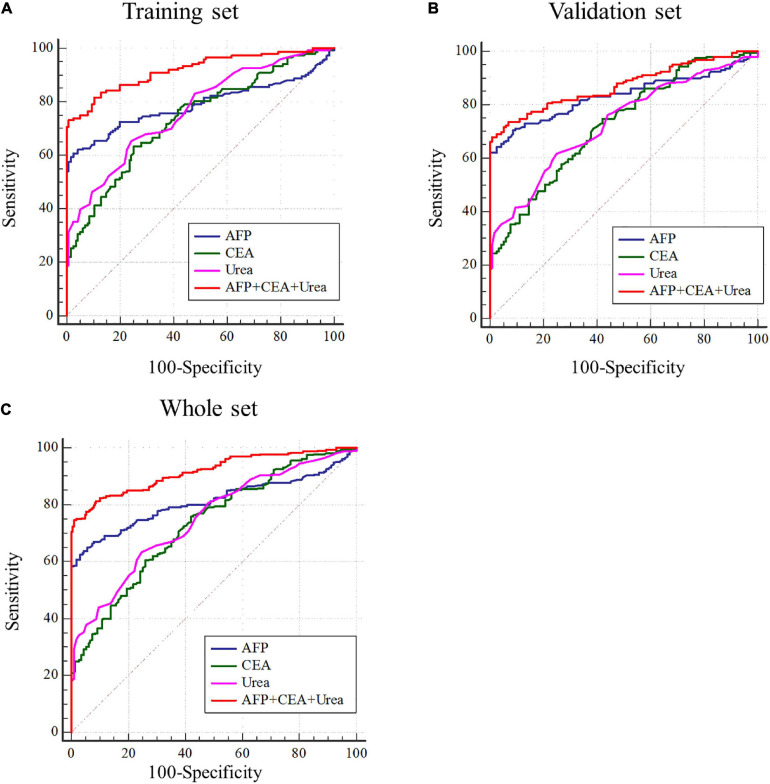
Performance of urea, AFP, and CEA in the diagnosis of HCC. **(A–C)** Receiver operating characteristic curves of urea, AFP, CEA and AFP + CEA + urea to distinguish HCC from healthy controls in training set, validation set and whole set.

AFP is closely related to the occurrence and development of liver cancer and a variety of tumors, mainly used as a serum marker for the diagnosis of primary liver cancer in clinical practice (Ahn et al., 2020; Su et al., 2020). Carcinoembryonic antigen (CEA) is a broad-spectrum tumor marker, which plays an important role in the differential diagnosis, disease monitoring, and efficacy evaluation of malignant tumors (Cheong et al., 2020; Chi et al., 2020). Performances of urea, AFP, and CEA as individual and combined HCC biomarkers were evaluated by the ROC curves. These three markers individually showed unsatisfactory performance for HCC diagnosis in the training set (AUC_*urea*_ = 0.766, AUC_*AFP*_ = 0.789, AUC_*CEA*_ = 0.736), the validation set (AUC_*urea*_ = 0.730, AUC_*AFP*_ = 0.834, AUC_*CEA*_ = 0.727), and the whole set (AUC_*urea*_ = 0.748, AUC_*AFP*_ = 0.812, AUC_*CEA*_ = 0.731). Nevertheless, combining the three indicators as a panel could significantly improve the diagnostic efficiency, with the AUC_*AFP+CEA+urea*_ = 0.918 in the training set, AUC_*AFP+CEA+urea*_ = 0.872 in the validation set, AUC_*AFP+CEA+urea*_ = 0.917 in the whole set (*P* < 0.05) (Figures 3A–C and Table 2).

**TABLE 2:** Performances of biomarkers for the diagnosis of HCC.

	AUC (95% CI)	Sensitivity (%)	Specificity (%)
**Training set**			
AFP	0.789 (0.735 to 0.836)	58.13	97.39
CEA	0.736 (0.678 to 0.787)	66.05	72.17
Urea	0.766 (0.713 to 0.817)	65.38	75.65
AFP + CEA + Urea	0.918 (0.879 to 0.948)*	73.20	99.13
**Validation set**			
AFP	0.834 (0.785 to 0.876)	60.98	100.00
CEA	0.727 (0.670 to 0.779)	75.00	57.38
Urea	0.730 (0.674 to 0.782)	61.64	75.00
AFP + CEA + Urea	0.872 (0.827 to 0.909)*	67.92	99.18
**Whole set**			
AFP	0.812 (0.777 to 0.844)	60.49	96.62
CEA	0.731 (0.692 to 0.768)	63.19	71.31
Urea	0.748 (0.709 to 0.784)	63.49	75.32
AFP + CEA + Urea	0.917 (0.891 to 0.939)*	74.68	99.13

*P < 0.05 in comparison with AFP, CEA or Urea, AUC, area under the curve; CI, confidence interval.

### The Key Enzymes of the Urea Cycle Were Often Low Expressed in HCC Patients

**FIGURE 4:**
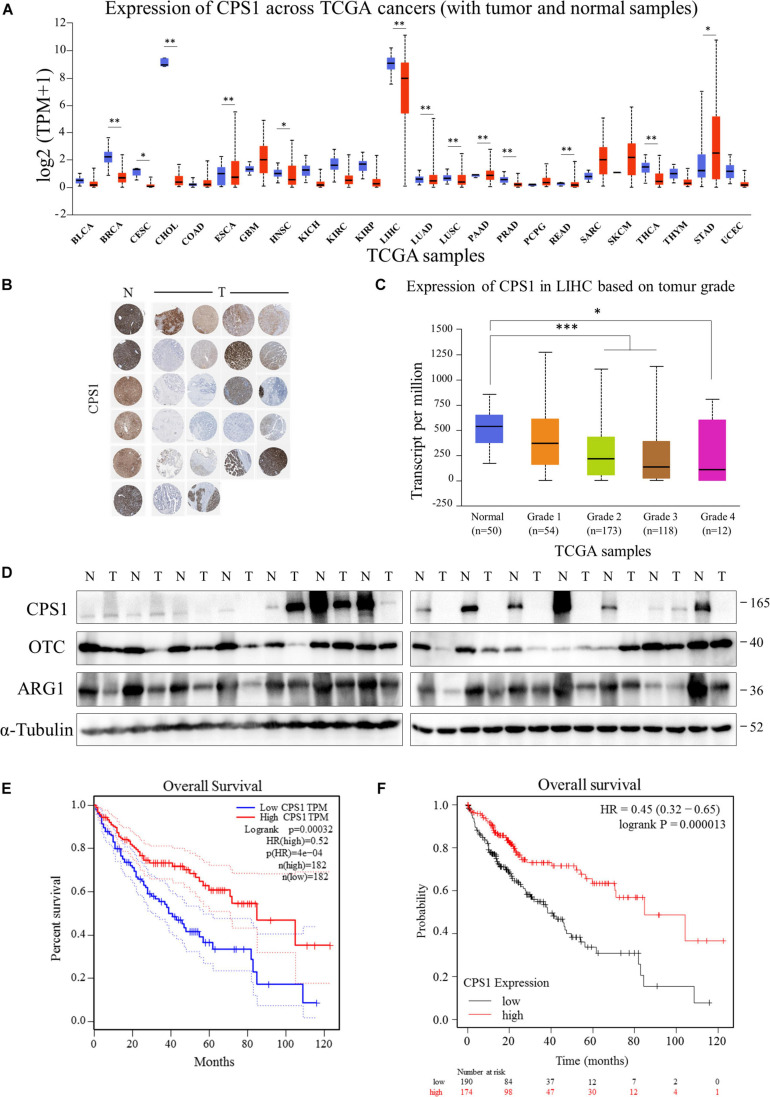
The key enzymes of the urea cycle were often low expressed in HCC patients. **(A)** UALCAN portal analysis of cancer samples from the TCGA database (http://ualcan.path.uab.edu/). A comparison of CPS1 expression between normal and multiple cancer samples. Tumor tissues were shown in red, and normal tissues were shown in blue. BLCA, Bladder urothelial carcinoma; BRCA, Breast invasive carcinoma; CESC, Cervical squamous cell carcinoma; CHOL, Cholangiocarcinoma; COAD, Colon adenocarcinoma; ESCA, Esophageal carcinoma; GBM, Glioblastoma multiforme; HNSC, Head and Neck squamous cell carcinoma; KICH, Kidney Chromophobe; KIRC, Kidney renal clear cell carcinoma; KIRP, Kidney renal papillary cell carcinoma; LIHC, Liver hepatocellular carcinoma; LUAD, Lung adenocarcinoma; LUSC, Lung squamous cell carcinoma; PAAD, Pancreatic adenocarcinoma; PRAD, Prostate adenocarcinoma; PCPG, Pheochromocytoma and Paraganglioma; READ, Rectum adenocarcinoma; SARC, Sarcoma; SKCM, Skin Cutaneous Melanoma; THYM, Thymoma; STAD, Stomach adenocarcinoma; UCEC, Uterine Corpus Endometrial Carcinoma. **(B)** Expression of CPS1 of normal liver (N) and HCC (T) samples from The Human Protein Atlas (Human Protein Atlas available from http://www.proteinatlas.org). **(C)** UALCAN portal analysis of CPS1 expression between normal and different grade HCC samples from the TCGA database. **(D)** Western blot to detect the expression of CPS1, OTC, ARG1 and GAPDH from 14 pairs of cancer and adjacent tissues of HCC. **(E,F)** Survival probability between HCC patients with high and low CPS1 expression. The GEPIA database (http://gepia.cancer-pku.cn/) and Kaplan-Meier Plotter (http://kmplot.com/analysis/) were used to conduct survival analyses based on core gene expression. ^∗^*p* < 0.05; ^∗∗^*p* < 0.01, ^∗∗∗^*p* < 0.001, ^****^p < 0.0001.

Next, we investigated the mechanism of elevated serum urea in cancer patients. Serum urea in patients with various tumors was significantly increased, we asked whether the ammonia released by the vigorous amino acid metabolism in cancer cells was detoxified to urea and excreted from the cells. However, by querying the UALCAN database (Chandrashekar et al., 2017), it was found to be inconsistent with our speculations. The key enzymes of the urea cycle, Carbamoyl-phosphate synthase 1 (CPS1), Ornithine carbamoyl-transferase (OTC), and Arginase (ARG1) were highly expressed in the liver, but normal tissues were significantly higher than cancer tissues in most cases (Figure 4A and Supplementary Figures 2A, 3A). Immunohistochemistry results from The Human Protein Atlas (Uhlén et al., 2015) showed that CPS1, OTC, and ARG1 were often low expressed in HCC tissue compared with normal liver tissue (Figure 4B and Supplementary Figures 2B, 3B). Moreover, as the tumor progressed, the expression of key enzymes of the urea cycle tended to gradually decrease in HCC (Figure 4C and Supplementary Figures 2C, 3C). Meanwhile, we collected 14 pairs of HCC and adjacent tissues to detect the expression of CPS1, OTC and ARG1. Our results showed that the expression of CPS1, OTC and ARG1 in most HCC tissues were lower than that in normal liver tissues. Surprisingly, CPS1 was not expressed in more than half of tested HCC tissues (Figure 4D). By querying the GEPIA and Kaplan-Meier Plotter database (Tang et al., 2017; Menyhárt et al., 2018), we found HCC patients with high expression of CPS1, OTC, and ARG1 had higher overall survival rate (Figures 4E,F and Supplementary Figures 2D,E, 3D,E), which might be due to the low expression of CPS1 in patients with advanced HCC. However, the mechanism of elevated urea remained to be determined.

### Ammonia Is Metabolized by Normal Liver and Cancer Cells Into Urea

**FIGURE 5:**
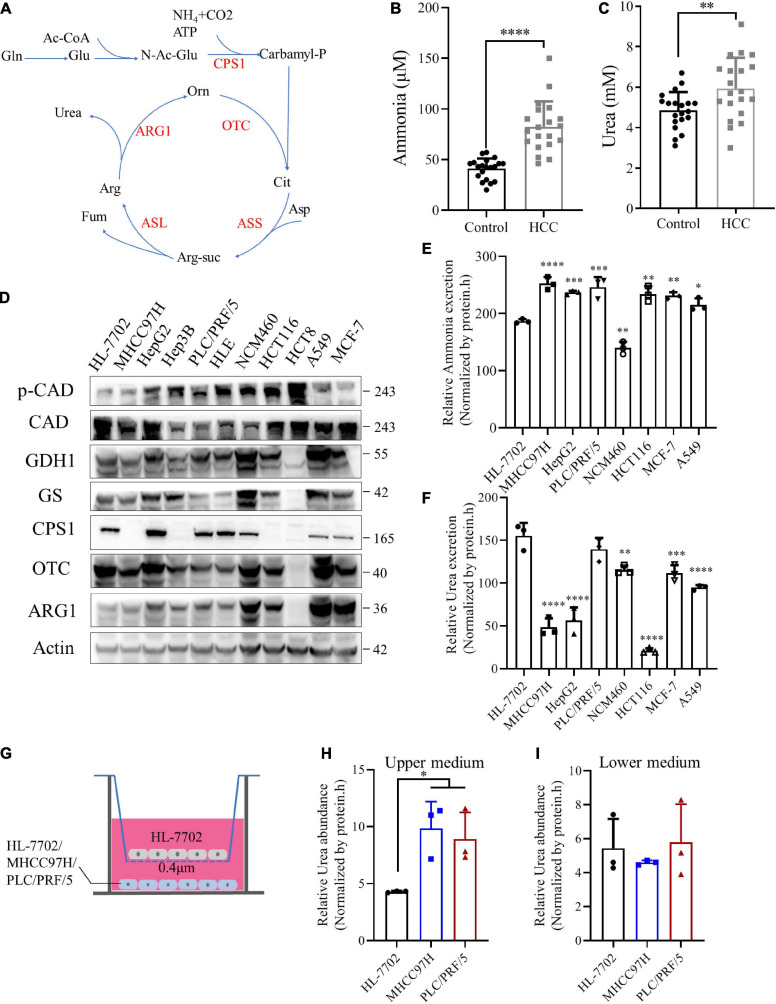
Ammonia is metabolized by normal liver and cancer cells into urea**. (A)** A schematic to show the metabolism of urea cycle. **(B)** Scatter plots with bar of plasma ammonia of 20 age and sex matched control and HCC patients. **(C)** Scatter plots with bar of serum urea of 20 age and sex matched control and HCC patients. **(D)** Western blot of lysates from HL-7702, MHCC97H, HepG2, Hep3B, PLC/PRF/5, HLE, NCM460, HCT116, HCT8, MCF-7 and A549 cells. **(E)** Excretion of ammonia to medium from HL-7702, MHCC97H, HepG2, PLC/PRF/5, NCM460, HCT116, MCF-7 and A549 cells. **(F)** Excretion of urea to medium from HL-7702, MHCC97H, HepG2, PLC/PRF/5, NCM460, HCT116, MCF-7 and A549 cells. **(G)** Schematic diagram of co-culture cell model. HL-7702, MHCC97H or PLC/PRF/5 were cultured in the lower layer respectively, and the upper layer was HL-7702. **(H)** Relative urea abundance in the upper medium of the cell lines as indicated. **(I)** Relative urea abundance in the lower medium of the cell lines as indicated. Values are the means ± SD of three independent experiments. ^∗^*p* < 0.05; ^∗∗^*p* < 0.01, ^∗∗∗^*p* < 0.001, ^****^p < 0.0001.

The urea cycle plays an important role in the protection against excess ammonia. The physiological concentration of ammonia in the plasma of healthy people ranges from 0–50 μM (Dasarathy et al., 2017). Super-physiological concentration of ammonia is toxic to neurons, and most studies believe that it is also toxic to cancer cells (Braissant, 2010; Kappler et al., 2017). Until Jessica B. et al. revealed that breast cancer cells could recycle ammonia for biosynthesis (Spinelli et al., 2017), but the fate of ammonia in other tumors has not been fully studied, we draw a schematic diagram of the urea cycle (Figure 5A). We speculated it might be HCC and other cancer cells release ammonia into the blood or microenvironment. Then normal liver cells metabolized it into urea and secreted to the blood.

To test this hypothesis, we collected plasma and serum samples from HCC patients and normal controls to detect ammonia and urea levels. Consistent with our speculation, the levels of ammonia and urea in HCC patients were significantly higher than healthy controls (Figures 5B,C), indicating ammonia in the microenvironment of HCC could be detoxified by urea. CCLE analysis (Ghandi et al., 2019) showed that CPS1 mRNA was expressed in some cancer cell lines, but most cancer cells did not express OTC and ARG1, implying the urea cycle was down-regulated in cancer cells (Supplementary Figure 4A). Next, we used normal and cancer cell lines for protein level verification, including normal liver cell HL-7702, HBV + HCC cell line PLC/PRF/5, Hep3B, HBV- HCC cell line HepG2, MHCC97H, and HLE, breast cancer cell MCF-7, lung cancer cell A549, normal colon cell line NCM460, colorectal cancer cell HCT116 and HCT8. Our results showed that except for HL-7702, some cancer cells also express CPS1, which in PLC/PRF/5, HepG2 and HLE were even higher than HL-7702 (Figure 5D). The CPS1 expression of normal colon cell line NCM460 is higher than the colon cancer cell lines HCT116 and HCT8 (Figure 5D), consistent with the tissue level data (Figure 4A). To our surprise, OTC and ARG1 were expressed in most cancer cells. Coupled with HCC and adjacent tissues data, ARG1 and OTC1 were also expressed in most HCC tissues (Figure 4D), suggesting that the synthesis of urea might marginally depend on the expression of CPS1 (Figure 5D). Besides, we detected the expression of two other enzymes involved in ammonia assimilation, glutamate dehydrogenase 1 (GDH1), and glutamine synthetase (GS), which were expressed in most cancer cells, except for colon cancer cell line HCT8, but the level of phosphorylated CAD was higher in HCT8 than other cells. CAD can also catalyze glutamine to carbamyl-phosphate, and be activated by phosphorylation (Robitaille et al., 2013). Furthermore, the excretion of ammonia and urea was detected in these cells. We found that normal liver cell HL-7702 released lower ammonia (Figure 5E) but excreted more urea than other cancer cells (Figure 5F). Besides, normal colon cell NCM460 released lower ammonia (Figure 5E) but excreted more urea than colorectal cancer cell HCT116. PLC/PRF/5, MCF-7, and A549 with CPS1 expression excreted more urea than MHCC97H and HCT116 (Figure 5F). However, it was strange that HepG2, with the highest expression of CPS1, did not secrete much urea, suggesting ammonia-derived carbamyl-phosphate catalyzed by CPS1 might support anabolism in HepG2 cells, such as pyrimidine or polyamine biosynthesis.

Enzymes involved in ammonia assimilation were generally low expressed in HCC tissues (Figure 4D and Supplementary Figure 4B), implying that the ammonia assimilation capacity of HCC tissue was lower than normal liver tissue. In order to further validate the mechanism, we cultured normal liver cell line HL-7702 and a mixture of normal liver cells and HCC cell line MHCC97H with the same total number under the same conditions (Supplementary Figure 4C). Our results showed that the urea in the mixed culture medium was higher than the normal liver cells (Supplementary Figure 4D). Next, we established a co-culture model of normal liver and HCC cells, using a small chamber with a pore size of 0.4 μm. Cells in this small chamber could not pass, but metabolites could exchange. We cultured HL-7702, MHCC97H and PLC/PRF/5 in the lower layer, and the same amount of HL-7702 in the upper chamber, then collected the upper and lower media (Figure 5G). Our data revealed that the urea in the upper medium of MHCC97H and PLC/PRF/5 were higher than HL-7702, but there was no difference in the lower medium (Figures 5H,I). Besides, there was no difference in ammonia levels in both the upper and lower medium (Supplementary Figures 4E,F). Furthermore, in order to explore the metabolic phenomenon *in vivo*, we detected urea and ammonia in HCC and adjacent tissues. Our results showed that urea in HCC tissue was lower than adjacent tissues, while ammonia in HCC tissues was higher than that in adjacent tissues (Supplementary Figures 4G,H). These data demonstrating the extra ammonia released by HCC cells could indeed be metabolized by normal liver cells into urea. Taken together, these data indicate that the ability to metabolize ammonia into urea in cancer cells marginally depends on the expression of CPS1. Normal liver and cancer cells cooperate to metabolize ammonia into urea: excessive ammonia from cancer cells can be metabolized into urea by normal liver cells and secreted into medium (blood), leading to the elevated serum urea in HCC patients.

### The Relationship of Urea Cycle With Ammonia Metabolism in Cancer Cells

**FIGURE 6:**
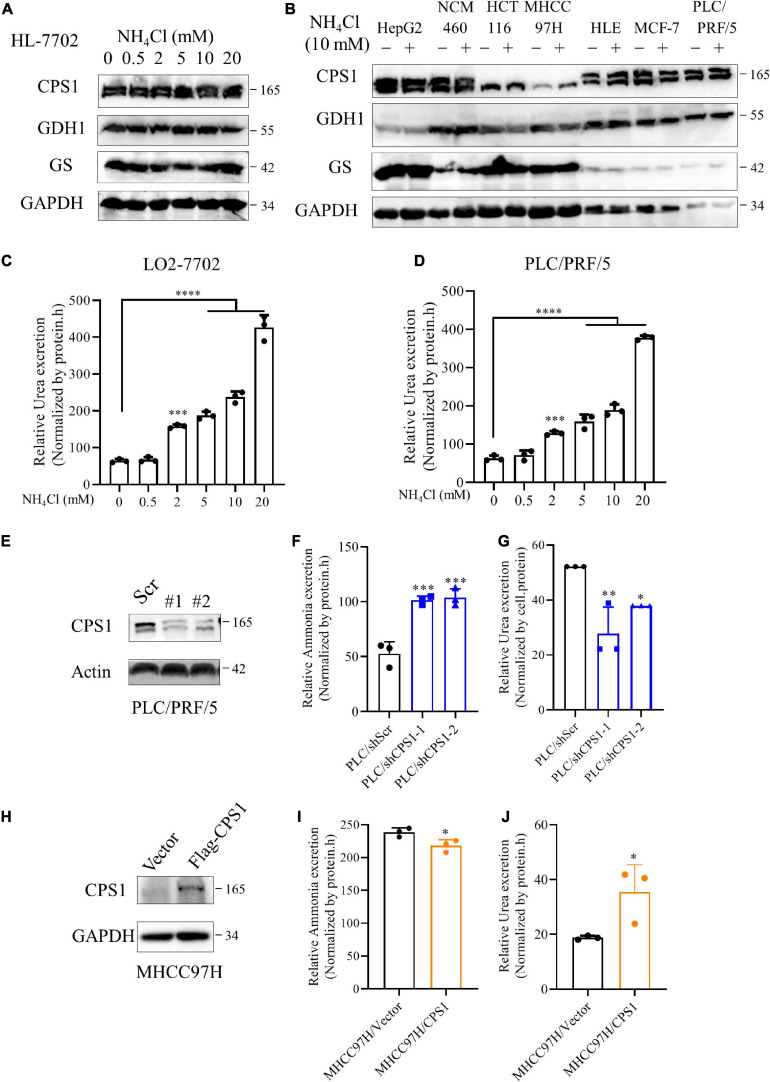
The relationship of urea cycle with ammonia metabolism in cancer cells. **(A)** Western blot of lysates from HL-7702 cells cultured under 0, 0.5, 2, 5, 10 or 20 mM NH_4_Cl for 8 h. **(B)** Western blot of lysates from HepG2, NCM460, HCT116, MHCC97H, HLE, MCF-7, PLC/PRF/5 cells cultured under 0 or 10 mM NH_4_Cl for 8 h. **(C,D)** Relative urea excretion from HL-7702 and PLC/PRF/5 cells cultured under 0, 0.5, 2, 5, 10 or 20 mM NH_4_Cl for 48 h. **(E)** Western blot confirmed the knockdown of CPS1 in PLC/PRF/5 cells. **(F)** Relative ammonia excretion in PLC/PRF/5/shScramble, PLC/PRF/5/shCPS1 for 8 hours. **(G)** Relative urea excretion in PLC/shScr, PLC/shCPS1 for 48 h. **(H)** Western blot confirmed the over-expression of CPS1 in MHCC97H cells. **(I)** Relative ammonia excretion in MHCC97H/Vector, MHCC97H/CPS1 for 8 h. **(J)** Relative urea excretion in MHCC97H/Vector, MHCC97H/CPS1 for 48 h. Values are the means ± SD of three independent experiments. ^∗^*p* < 0.05; ^∗∗^*p* < 0.01, ^∗∗∗^*p* < 0.001, ^****^p < 0.0001.

To further investigate this interesting phenomenon, we treated cells with ammonium chloride (NH_4_Cl) and detected the expression of enzymes involved in ammonia assimilation. To our disappointment, the expression of CPS1, GDH1, and GS was not enhanced under various concentrations of ammonia in HL-7702 (Figure 6A). Because 10 mM NH_4_Cl does not significantly affect the pH of the culture medium (Spinelli et al., 2017), we treated cancer cells with 10 mM NH_4_Cl, the expression of enzymes involved in ammonia assimilation was not significantly increased either (Figure 6B). Jessica B. et al. (Spinelli et al., 2017) reported ammonia enhances GDH activity to support biosynthesis, Hakvoort et al. showed the detoxification of stepwise increments of intravenously infused ammonia depends on GS activity (Hakvoort et al., 2017), suggesting ammonia might affect the activity of CPS1, GDH1 and GS. Next, we treated normal liver cell HL-7702 and HCC cell line PLC/PRF/5 with different concentrations of NH_4_Cl and detect urea secretion. Our results showed that ammonia induced concentration-dependent urea excretion in HL-7702 and PLC/PRF/5 cells (Figures 6C,D). Then we knocked down the CPS1 in HCC cell lines PLC/PRF/5 and HepG2(Figure 6E and Supplementary Figure 5A). Knockdown of CPS1 increased the release of ammonia and suppressed the excretion of urea in PLC/PRF/5 and HepG2 cells (Figures 6F,G and Supplementary Figures 5B,C). Furthermore, we over-expressed CPS1 in MHCC97H and HCT116. MHCC97H and HCT116 cells with CPS1 over-expression secreted more urea but less ammonia (Figures 6H–J and Supplementary Figures 5D–F). The above results indicate that urea cycle can detoxify high concentrations of ammonia.

### Urea Cycle Protects Cancer Cells From the High Concentration of Ammonia

**FIGURE 7:**
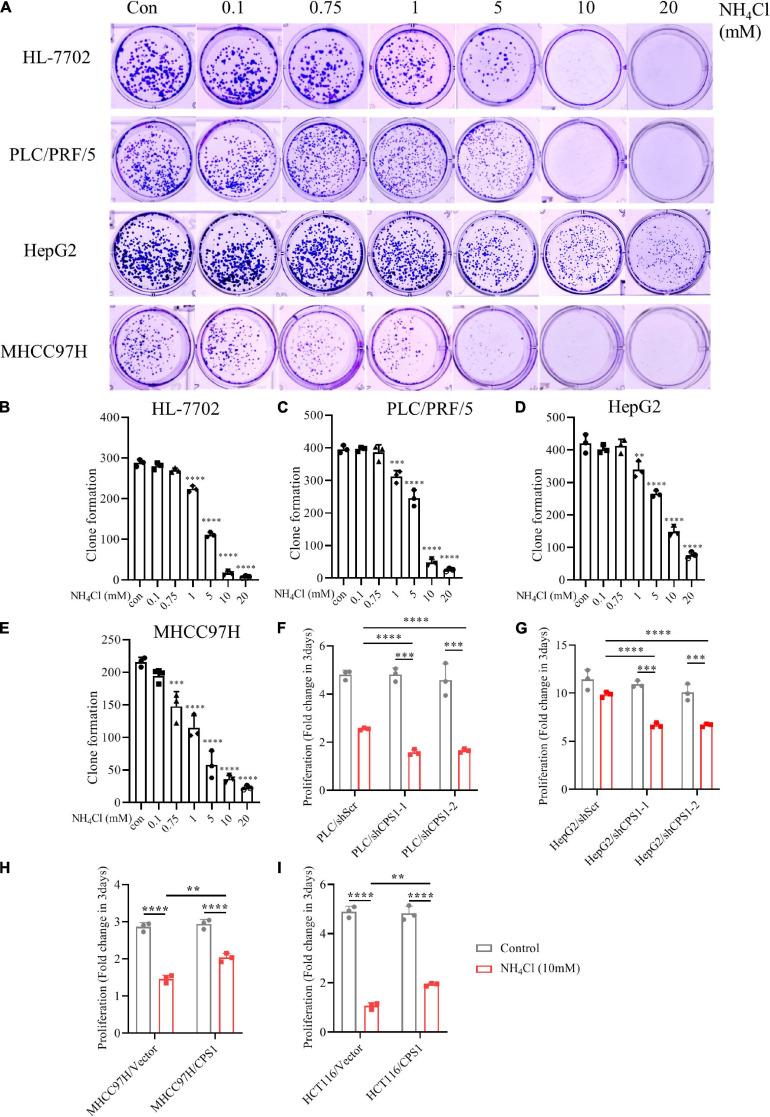
Urea cycle protects cancer cells from high concentrations of ammonia. **(A)** Colony formation ability of HL-7702, PLC/PRF/5, HepG2 and MHCC97H under different concentrations of NH_4_Cl. The culture time was 2 weeks. **(B–E)** Colony number quantification of HL-7702, PLC/PRF/5, HepG2 and MHCC97H under different concentrations of NH_4_Cl by Image J. **(F)** Proliferation of PLC/PRF/5/shScr and PLC/PRF/5/shCPS1 cells cultured under control or 10 mM NH_4_Cl for 3 days. **(G)** Proliferation of HepG2/shScr and HepG2/shCPS1 cells cultured under control or 10 mM NH_4_Cl for 3 days. **(H)** Proliferation of MHCC97H/Vector, MHCC97H/CPS1 cells cultured under control or 10 mM NH_4_Cl for 3 days. **(I)** Proliferation of HCT116/Vector, HCT116/CPS1 cells cultured under control or 10 mM NH_4_Cl for 3 days. Values are the means ± SD of three independent experiments. ^∗^*p* < 0.05; ^∗∗^*p* < 0.01, ^∗∗∗^*p* < 0.001, ^****^p < 0.0001.

To investigate whether ammonia was metabolized into urea or contributed to biosynthesis, we observed the effect of different concentrations of NH_4_Cl on cancer cell colony formation. The number of colonies of MHCC 97H and HCT116 cells with low expression of CPS1 decreased significantly at the concentration of 0.75 mM NH_4_Cl, but the number of colonies of cells with high expression of CPS1 begun to decline at 1mM. Cells with high CPS1 expression formed more colonies than cells with low expression of CPS1 under high concentrations of NH_4_Cl (5-10 mM) (Figures 7A–E and Supplementary Figures 6A–D). The number of clones formed by MCF-7 even begun to decrease until 5mM NH_4_Cl treatment (Supplementary Figures 6A,D), might mainly because breast cancer cells can recycle ammonia through GDH (Spinelli et al., 2017). Then we treated PLC/PRF/5 and HepG2 cells with high concentrations of NH_4_Cl and detected the proliferation. 5 mM NH_4_Cl could inhibit the proliferation of PLC/PRF/5, but HepG2 could even tolerate ultra-high concentration of ammonia (20 mM) without significantly affecting its proliferation (Supplementary Figures 7A,B). This might be due to the high expression of CPS1 in HepG2, but its urea excretion was not high (Figure 5F), indicating ammonia might have other metabolic fate in HepG2 cells. Study also showed that ammonia via urea cycle contributes to polyamine biosynthesis and promotes tumor growth (Li et al., 2019). Next, we used 10 mM NH_4_Cl to treat PLC/PRF/5 and HepG2 cells with CPS1 down-regulation to observe the role of urea cycle in proliferation. Our results showed that knockdown of CPS1 does not significantly inhibit cancer cell proliferation, but can make cancer cells more sensitive to ammonia toxicity (Figures 7F,G). Moreover, knockdown of CPS1 did not affect cell colony formation under normal condition, but suppressed the ability of clone formation of cancer cells under 10 mM of NH_4_Cl (Supplementary Figures 7A–C). Furthermore, MHCC97H and HCT116 cells with CPS1 over-expression could tolerate higher ammonia toxicity than controls (Figures 7H,I and Supplementary Figures 7D–F). Taken together, these results suggest that in addition to biosynthesis, cancer cells metabolized a part of ammonia into a non-toxic form and secreted out of cells, and released excess ammonia out of cells into the blood, which was finally metabolized into urea by normal liver cells.

## Discussion

The work presented here was undertaken to investigate new serum biomarkers for HCC by targeted quantitative metabolomics and automated biochemical analyzer. Our results revealed that urea was a potential serum biomarker for HCC, and could be used to distinguish HCC from other liver diseases. Urea combined with AFP and CEA can significantly improve the diagnostic efficiency of HCC. But the key enzymes of the urea cycle, especially CPS1, were often low expressed in HCC and other cancer tissues. Strikingly, we found the plasma ammonia in HCC patients was significantly higher than normal people. Moreover, cell experiments showed that normal liver cells released lower ammonia but excreted more urea than cancer cells. The urea metabolism in cancer cells might marginally depend on the expression of CPS1. Our co-culture cell models further revealed that excess ammonia from HCC cells can be metabolized into urea by normal liver cells. But the expression of CPS1 was not enhanced with the concentration of NH_4_Cl; it might regulate the urea cycle through enzyme activity. Furthermore, the urea cycle could detoxify high concentrations of ammonia to promote cancer cell proliferation. This study demonstrated the role of urea cycle in cancer cells and the possibility of urea as a serum biomarker for HCC.

Identifying accurate non-invasive biomarkers that can be widely used remains a challenge. To date, many plasma/serum metabolic biomarkers have been reported for the early diagnosis of HCC (Banales et al., 2019; Han et al., 2020). However, few biomarkers have been used in the clinical diagnosis of HCC, mainly because most clinical laboratories cannot detect them. In our study, we found that serum urea, a common clinical indicator, was significantly higher in HCC patients than healthy controls and other liver diseases. Moreover, the serum urea of patients with lung cancer, breast cancer, and colorectal cancer were also significantly higher. Serum AFP is widely recognized and used for HCC diagnosis (Montal et al., 2019). However, it is difficult to characterize HCC with a single biomarker, since it is a complex disease caused by various risk factors with multiple pathogenic mechanisms (Galle and Foerster, 2019). Our data showed the sensitivity of AFP for HCC diagnosis is unsatisfactory (60.49%). But combined AFP with urea and CEA as a panel can significantly improve the diagnostic performance, leading to more early diagnosis of HCC.

The urea cycle can eliminate excess nitrogen and ammonia produced by protein decomposition or nitrogen compound synthesis in the human body (Caldwell et al., 2018; Lee et al., 2018). Urea cycle enzymes also contribute to anabolism, such as nucleotide biosynthesis in certain tumor types (Kim et al., 2017; Rom et al., 2018). However, the urea cycle dysregulation is a common feature of tumors (Lee et al., 2018). And the expression of key enzymes CPS1, OTC, ARG1 in the urea cycle were lower than normal tissue in HCC and other cancers. The HCC microenvironment is composed of stromal cells, hepatic stellate cells, endothelial cells and immune cells (Biswas et al., 2013). Crosstalk between cancer cells and their surrounding microenvironment is necessary to maintain HCC development by promoting angiogenesis, EMT or regulating the polarization of immune cells (Hernandez-Gea et al., 2013). From this, we speculate that it may be the metabolic process of HCC and other cancer cells release ammonia into the blood and/or microenvironment; normal liver cells detoxify ammonia into urea and secrete it into the serum to avoid the accumulation of ammonia in cancer cells. Because ammonia entry was regulated by diffusion, and ammonia can diffuse across the plasma membrane (Spinelli et al., 2017). Consistent with our speculation, the plasma ammonia in HCC patients was significantly higher than normal controls. Although we found cancer cells also excrete urea, most cancer cells excreted much less urea than normal liver cells. Besides, KEGG analysis of metabolomics revealed that the purine metabolism pathway in HCC patients was enriched. Skeletal muscle, myocardium, liver, and brain may all be deaminated by purine nucleotide cycles. Experiments have shown that 50% of the ammonia in brain tissue is produced by purine nucleotide cycles (Schultz and Lowenstein, 1978). Although some breast cancer cells can recycle ammonia for biosynthesis (Spinelli et al., 2017), our previous study proved that the reuse of ammonia by cancer cells is not universal: cancer cells excrete dihydroorotate out of the cells to avoid ammonia accumulation under hypoxia (Wang et al., 2019). Moreover, we found urea excretion marginally depended on the expression of CPS1 in cancer cells. Although the expression of CPS1 in HepG2 was much higher than HL-7702, HepG2 secreted less urea, indicating other metabolic fate of ammonia in HepG2. In addition to polyamine biosynthesis, it was also reported that concurrent occurrence of oncogenic KRAS and loss of LKB1 (KL) in cells the carbamoyl-phosphate catalyzed by CPS1 could be used for pyrimidine synthesis to maximize nitrogen utilization (Kim et al., 2017). Furthermore, we proved the underlying mechanism by direct contact cell model and co-culture cell models: normal liver cells can indeed metabolize the excess ammonia released by HCC cells, which may be the reason for the elevated serum urea in HCC patients. Moreover, the urea in HCC tissues was lower than that in adjacent tissues, while ammonia was higher, which further supports our speculation.

The ammonia-clearing system also includes GS and GDH1. However, the expression of CPS1, GS and GDH1 did not respond to high concentrations of NH_4_Cl in normal liver cells and cancer cells, consistent with Jessica B. et al. observations (Spinelli et al., 2017). They revealed breast cancer cells can assimilate ammonia through reductive amination catalyzed by GDH, but its expression was not changed under high concentrations of NH_4_Cl. CPS1 can be allosterically activated by N-acetyl-glutamate. We speculate that it may be high concentrations of ammonia accelerated the transamination reaction and cause the synthesis of N-acetyl-glutamate to enhance, thereby activating CPS1. Jessica B. et al. proved NH_4_Cl was not toxic to breast cancer cells (Spinelli et al., 2017). We indeed found MCF-7 could even tolerate 1 mM NH_4_Cl to proliferate. The cell lines with high expression of CPS1, including HL-7702, PLC/PRF/5, HepG2, and NCM460, could tolerate 0.75 mM NH_4_Cl without colony number decrease, but the number of colonies formed in cell lines with low CPS1 expression, including MHCC97H and HCT116 had begun to decline significantly under 0.75 mM NH_4_Cl. Knockdown of CPS1 did not inhibit the proliferation of PLC/PRF/5 and HepG2, which might be due to the compensation role of GS and/or GDH1. However, knockdown of CPS1 sensitizes PLC/PRF/5 and HepG2 to NH_4_Cl, which significantly suppressed their proliferation and robustly inhibited their colony formation. Besides, over-expression of CPS1 could improve the ability of MHCC97H and HCT116 to tolerate ammonia. Taken together, our results revealed that the urea cycle can detoxify high concentration of ammonia to promote the rapid proliferation of cancer cells. The ammonia metabolism in cancer cells might be highly efficient, a part of ammonia in cancer cells may be used for biosynthesis, but a large amount or excess ammonia needs to bemetabolized into non-toxic products by the cooperation of normal liver cells and cancer cells, and then secreted out of the cells. Winter M C et al. also reported that raised serum urea predicts for early death in small cell lung cancer (Winter et al., 2008). Next, we will investigate the effect of simultaneously inhibiting the three key enzymes in the ammonia-clearing system on cancer cell proliferation, and explore the possibility of them as targets for combination therapy.

In conclusion, the combination of metabolomics and routine testing can reveal the potential serum biomarkers of HCC, and quickly promote the application. Urea is a potential biomarker of HCC, and combining with AFP and CEA can improve the diagnostic efficiency of HCC. In addition to ammonia for biosynthesis, cancer cells also need to detoxify excess ammonia. Normal liver cells and cancer cells cooperate to metabolize ammonia into a non-toxic form, thereby promoting the proliferation of cancer cells.

## Data Availability Statement

The original contributions presented in the study are included in the article/Supplementary Material, further inquiries can be directed to the corresponding author/s.

## Ethics Statement

The studies involving human participants were reviewed and approved by Ethics Committee of Tianjin Medical University Cancer Institute and Hospital. The patients/participants provided their written informed consent to participate in this study.

## Author Contributions

CB concepted and designed the study and wrote the manuscript. HW performed sample collection for metabolomics testing and analysis. DD, TL, ZY, DL, WZ, RY, and LW collected samples and conducted biochemical testing. JG and ZW performed bioinformatics analysis. CB and HW performed cell experiments. LR and YL provided insightful ideas and guided the experiment process. All authors have read and agreed to the published version of the manuscript.

## Conflict of Interest

The authors declare that the research was conducted in the absence of any commercial or financial relationships that could be construed as a potential conflict of interest.

## Supplementary Material

Supplementary Figure 1 MetaboAnalyst analysis on altered metabolic pathways in HCC and HBV patients. **(A)** Results from MetaboAnalyst showing altered metabolic pathways in HCC patients versus healthy controls. Pathways are labeled: Arginine biosynthesis (1), Purine metabolism (2), Alanine, aspartate and glutamate metabolism (3), D-Glutamine and D-glutamate metabolism (4), Pyruvate metabolism (5), Citrate cycle (TCA cycle) (6), Cysteine and methionine metabolism (7), Arginine and proline metabolism (8). **(B)** Results from MetaboAnalyst showing altered metabolic pathways in HBV patients versus healthy controls. Pathways are labeled: Arginine biosynthesis (1), Citrate cycle (TCA cycle) (2), Pyruvate metabolism (3), Alanine, aspartate and glutamate metabolism (4), Phenylalanine, tyrosine and tryptophan biosynthesis (5), D-Glutamine and D-glutamate metabolism (6), Arginine and proline metabolism (7), Cysteine and methionine metabolism (8), Phenylalanine metabolism (9).

Supplementary Figure 2 Expression and prognosis analysis of OTC in HCC patients. **(A)** UALCAN portal analysis of cancer samples from the TCGA database (http://ualcan.path.uab.edu/). A Comparison of OTC expression between normal and multiple cancer samples. Tumor tissues were shown in red, and normal tissues were shown in blue. **(B)** Expression of OTC of normal and HCC samples from proteinatlas (Human Protein Atlas available from http://www.proteinatlas.org). **(C)** UALCAN portal analysis of OTC expression between normal and different grade HCC samples from the TCGA database. **(D,E)** Survival probability between HCC patients with high and low OTC expression. The GEPIA database (http://gepia.cancer-pku.cn/) and Kaplan-Meier Plotter (http://kmplot.com/analysis/) were used to conduct survival analyses based on core gene expression. **p* < 0.05; ***p* < 0.01, ****p* < 0.001, *****p* < 0.0001.

Supplementary Figure 3 Expression and prognosis analysis of ARG1 in HCC patients. **(A)** UALCAN portal analysis of cancer samples from the TCGA database (http://ualcan.path.uab.edu/). A comparison of ARG1 expression between normal and multiple cancer samples. Tumor tissues were shown in red, and normal tissues were shown in blue. **(B)** Expression of ARG1 of normal and HCC samples from proteinatlas (Human Protein Atlas available from http://www.proteinatlas.org). **(C)** UALCAN portal analysis of ARG1 expression between normal and different grade HCC samples from the TCGA database. **(D,E)** Survival probability between HCC patients with high and low ARG1 expression. The GEPIA database (http://gepia.cancer-pku.cn/) and Kaplan-Meier Plotter (http://kmplot.com/analysis/) were used to conduct survival analyses based on core gene expression. **p* < 0.05; ***p* < 0.01, ****p* < 0.001, *****p* < 0.0001.

Supplementary Figure 4 Ammonia is metabolized by normal liver and cancer cells into urea. **(A)** The mRNA expression of CPS1, OTC and ARG1 of cancer cells from CCLE database (https://portals.broadinstitute.org/ccle/about). **(B)** Western blot to detect the expression of GDH1, GS, and a-tubulin from 14 pairs of cancer and adjacent tissues of HCC. **(C)** Schematic diagram of cell model. 200, 000 HL7702 or 100, 000 HL-7702 and 100, 000 MHCC97H were cultured separately. **(D)** Relative urea excretion of the cell lines as indicated. **(E)** Relative ammonia abundance in the upper medium of the cell lines as indicated. **(F)** Relative ammonia abundance in the lower medium of the cell lines as indicated. **(G)** Relative urea abundance in 10 pairs of fresh HCC and adjacent tissues. **(H)** Relative ammonia abundance in 10 pairs of fresh HCC and adjacent tissues.

Supplementary Figure 5 The relationship of urea cycle with ammonia metabolism in cancer cells. **(A)** Western blot confirmed the knockdown of CPS1 in HepG2 cells. **(B)** Relative ammonia excretion in HepG2/Scramble, HepG2/shCPS1 for 8 h. **(C)** Relative urea excretion in HepG2/Scramble, HepG2/shCPS1 for 48 h. **(D)** Western blot confirmed the over-expression of CPS1 in HCT116 cells. **(E)** Relative ammonia excretion in HCT116/Vector, HCT116/CPS1 for 8 h. **(F)** Relative urea excretion in HCT116/Vector, HCT116/CPS1 for 48 h. Values are the means ± SD of three independent experiments. **p* < 0.05, ***p* < 0.01, ****p* < 0.001.

Supplementary Figure 6 High concentrations of ammonia inhibits the proliferation of cancer cells. **(A)** Colony formation ability of NCM460, HCT116 and MCF-7 under different concentrations of NH_4_Cl. **(B–D)** Colony number quantification of NCM460, HCT116, and MCF-7 under different concentrations of NH_4_Cl by Image J. **(E,F)** Proliferation of PLC/PRF/5 and HepG2 cells cultured under 0, 5, 10 or 20 mM NH_4_Cl for 3 days. Values are the means ± SD of three independent experiments. **p* < 0.05; ***p* < 0.01, ****p* < 0.001, *****p* < 0.0001.

Supplementary Figure 7 Urea cycle protect cancer cells from high concentrations of ammonia. **(A)** Colony formation ability of PLC/PRF/5/shScr and PLC/PRF/5/shCPS1-1, HepG2/shScr and HepG2/shCPS1-1 under control or 10 mM NH_4_Cl. **(B,C)** Colony number quantification of PLC/PRF/5/shScr and PLC/PRF/5/shCPS1-1, HepG2/shScr and HepG2/shCPS1-1 under control or 10mM NH_4_Cl by Image J. **(D)** Colony formation ability of MHCC97H/Vector and MHCC97H/CPS1, HCT116/Vector and HCT116/CPS1 under control or 10mM NH_4_Cl. **(E,F)** Colony number quantification of MHCC97H/Vector and MHCC97H/CPS1, HCT116/Vector and HCT116/CPS1 under control or 10 mM NH_4_Cl by Image J. Values are the means ± SD of three independent experiments. **p* < 0.05; ***p* < 0.01, ****p* < 0.001, *****p* < 0.0001.

Supplementary Table 1 Characteristics of serum samples from healthy controls, HBV, HBV positive cirrhosis, HCV, HBV positive HCC patients, and metastatic HCC patients.

Supplementary Table 2 Characteristics of serum samples from lung cancer, breast cancer, colorectal cancer, HCC patients and healthy controls.
