# Supplementary figures and images for: Urea as a By-Product of Ammonia Metabolism Can Be a Potential Serum Biomarker of Hepatocellular Carcinoma
Source: Front Cell Dev Biol. 2021 Apr 1;9:650748. doi: 10.3389/fcell.2021.650748 (PMC8047217; doi:10.3389/fcell.2021.650748)

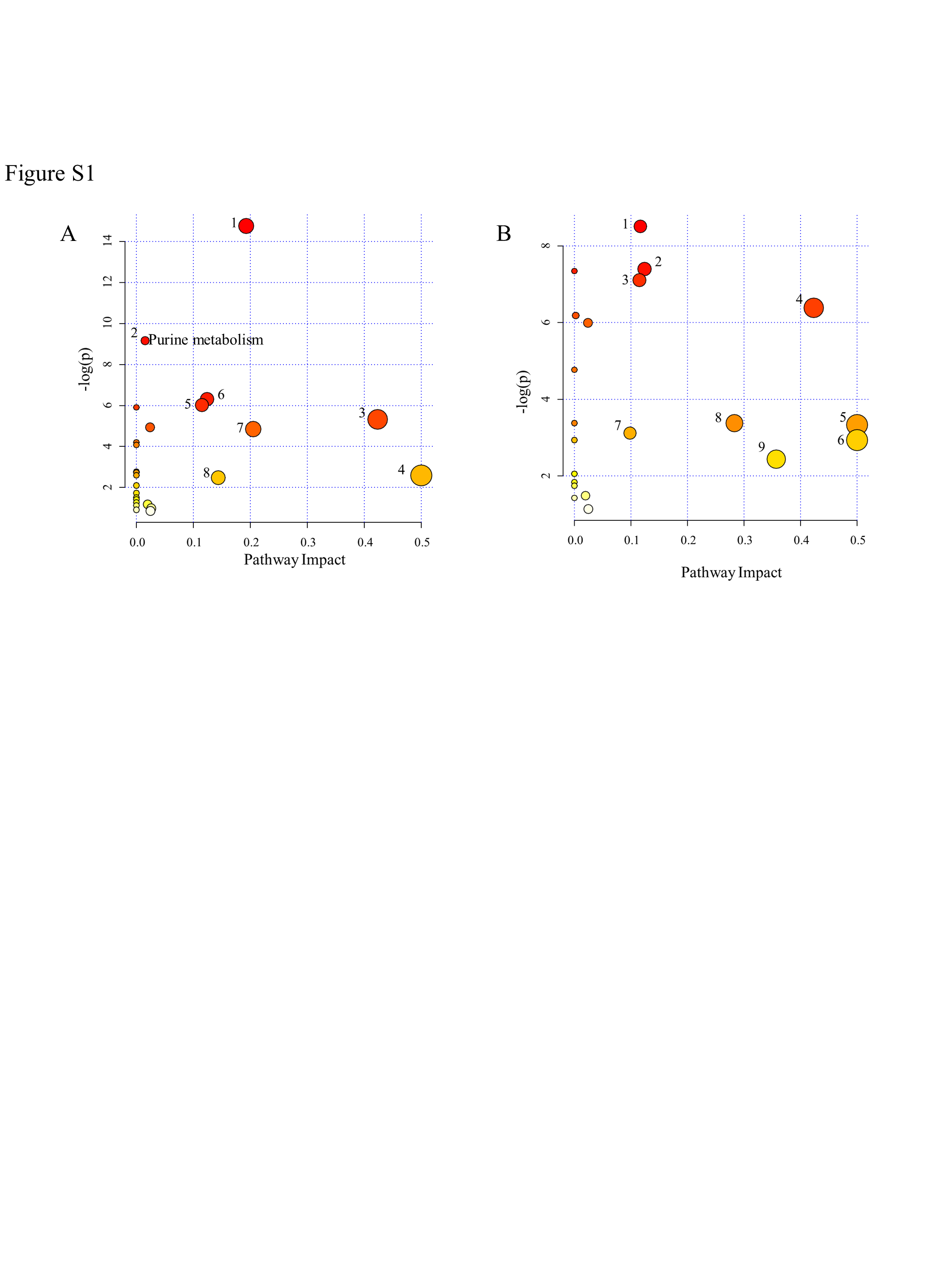

Supplement: Supplementary Figure 1 — MetaboAnalyst analysis on altered metabolic pathways in HCC and HBV patients. (A) Results from MetaboAnalyst showing altered metabolic pathways in HCC patients versus healthy controls. Pathways are labeled: Arginine biosynthesis (1), Purine metabolism (2), Alanine, aspartate and glutamate metabolism (3), D-Glutamine and D-glutamate metabolism (4), Pyruvate metabolism (5), Citrate cycle (TCA cycle) (6), Cysteine and methionine metabolism (7), Arginine and proline metabolism (8). (B) Results from MetaboAnalyst showing altered metabolic pathways in HBV patients versus healthy controls. Pathways are labeled: Arginine biosynthesis (1), Citrate cycle (TCA cycle) (2), Pyruvate metabolism (3), Alanine, aspartate and glutamate metabolism (4), Phenylalanine, tyrosine and tryptophan biosynthesis (5), D-Glutamine and D-glutamate metabolism (6), Arginine and proline metabolism (7), Cysteine and methionine metabolism (8), Phenylalanine metabolism (9). [file Image_1.TIF]

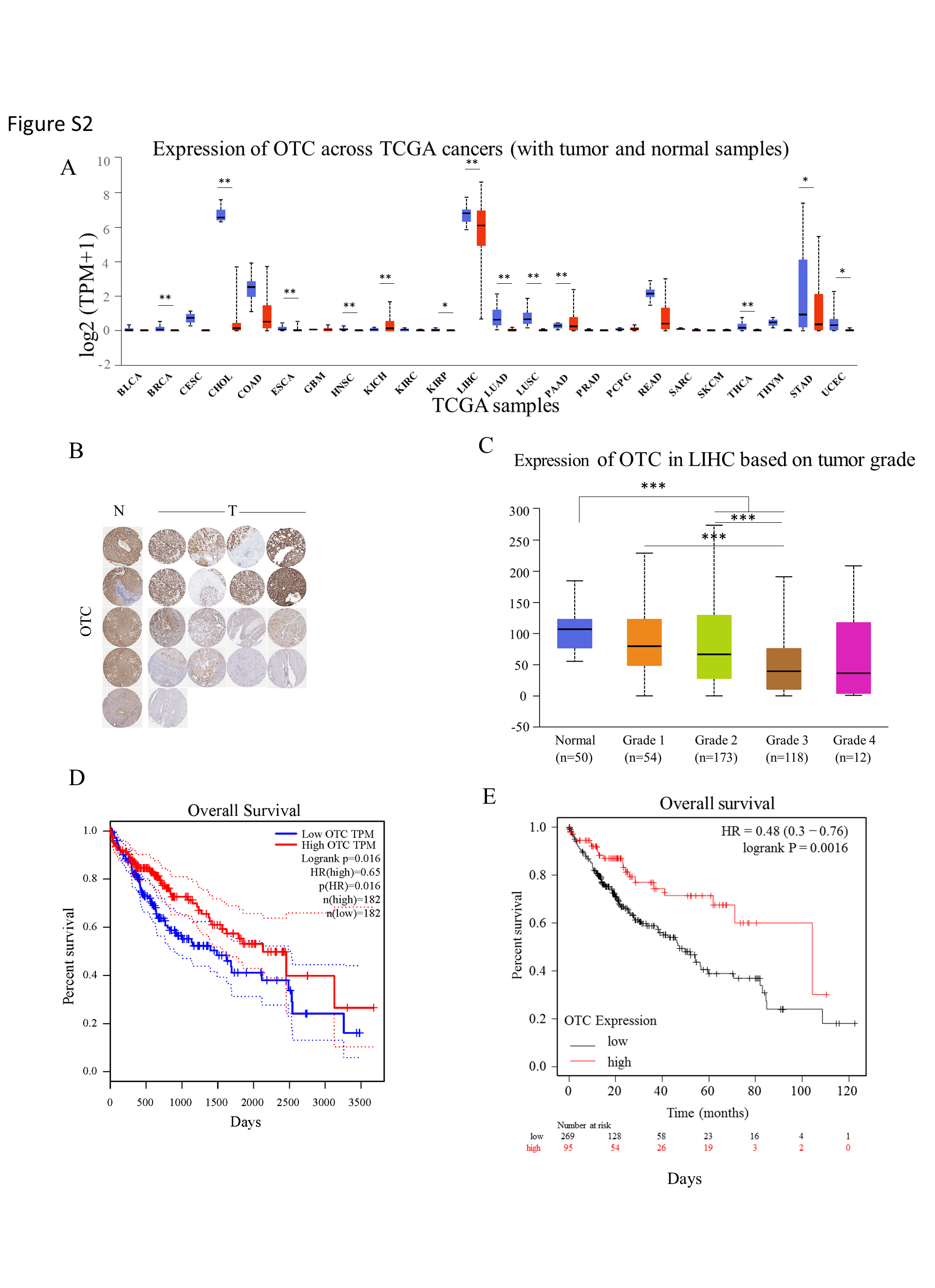

Supplement: Supplementary Figure 2 — Expression and prognosis analysis of OTC in HCC patients. (A) UALCAN portal analysis of cancer samples from the TCGA database (http://ualcan.path.uab.edu/). A Comparison of OTC expression between normal and multiple cancer samples. Tumor tissues were shown in red, and normal tissues were shown in blue. (B) Expression of OTC of normal and HCC samples from proteinatlas (Human Protein Atlas available from http://www.proteinatlas.org). (C) UALCAN portal analysis of OTC expression between normal and different grade HCC samples from the TCGA database. (D,E) Survival probability between HCC patients with high and low OTC expression. The GEPIA database (http://gepia.cancer-pku.cn/) and Kaplan-Meier Plotter (http://kmplot.com/analysis/) were used to conduct survival analyses based on core gene expression. *p < 0.05; **p < 0.01, ***p < 0.001, ****p < 0.0001. [file Image_2.tif]

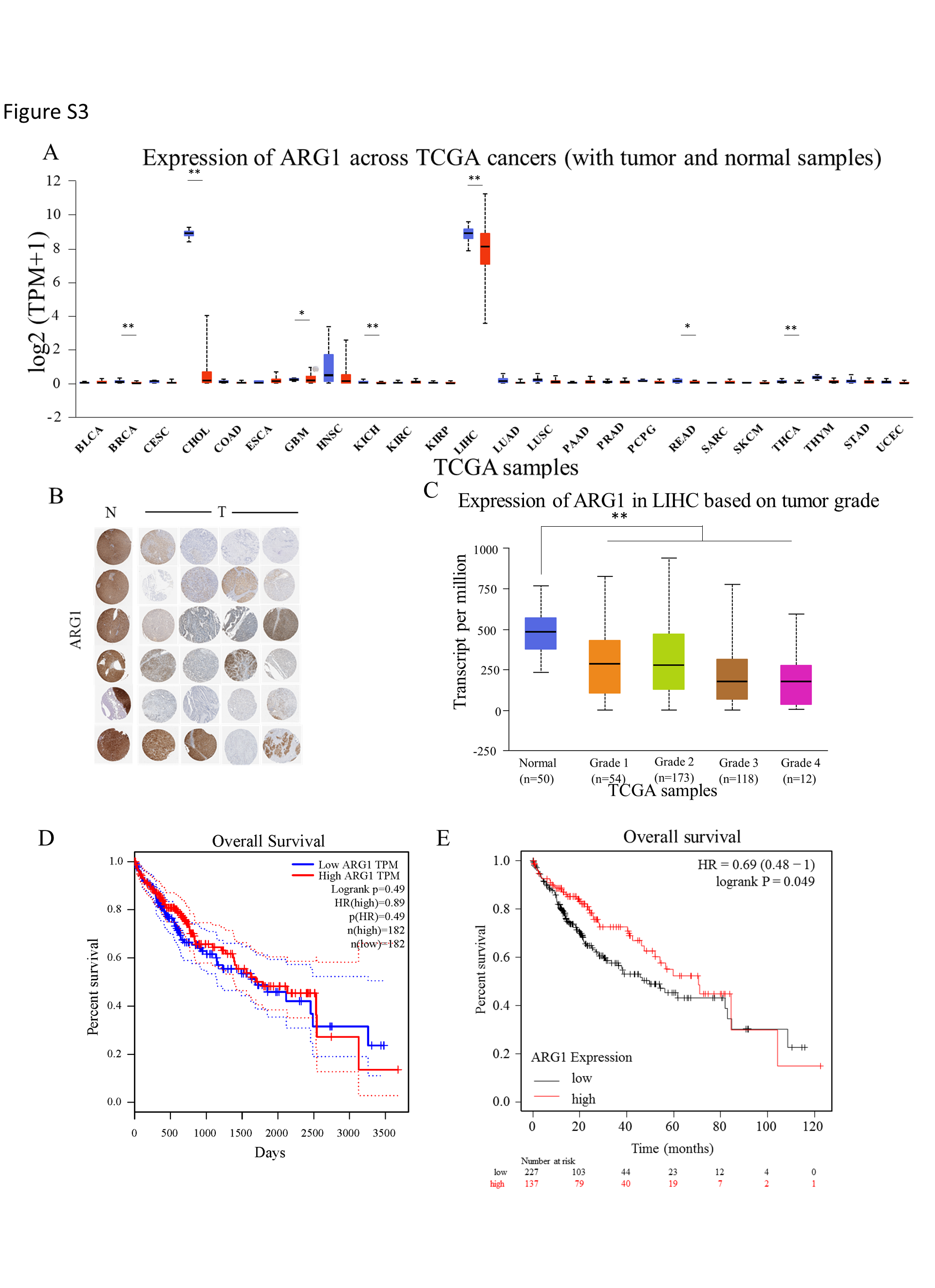

Supplement: Supplementary Figure 3 — Expression and prognosis analysis of ARG1 in HCC patients. (A) UALCAN portal analysis of cancer samples from the TCGA database (http://ualcan.path.uab.edu/). A comparison of ARG1 expression between normal and multiple cancer samples. Tumor tissues were shown in red, and normal tissues were shown in blue. (B) Expression of ARG1 of normal and HCC samples from proteinatlas (Human Protein Atlas available from http://www.proteinatlas.org). (C) UALCAN portal analysis of ARG1 expression between normal and different grade HCC samples from the TCGA database. (D,E) Survival probability between HCC patients with high and low ARG1 expression. The GEPIA database (http://gepia.cancer-pku.cn/) and Kaplan-Meier Plotter (http://kmplot.com/analysis/) were used to conduct survival analyses based on core gene expression. *p < 0.05; **p < 0.01, ***p < 0.001, ****p < 0.0001. [file Image_3.tif]

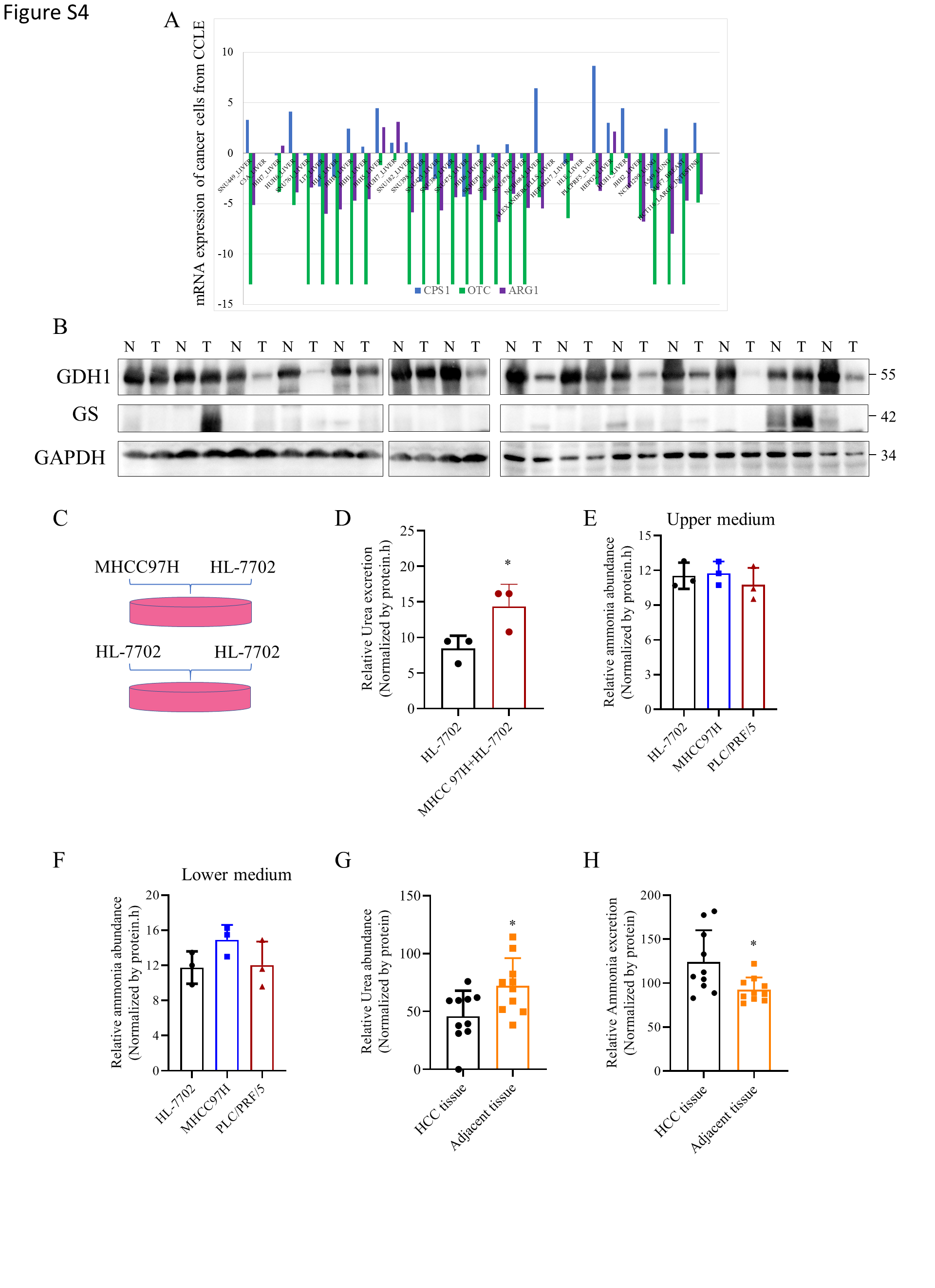

Supplement: Supplementary Figure 4 — Ammonia is metabolized by normal liver and cancer cells into urea. (A) The mRNA expression of CPS1, OTC and ARG1 of cancer cells from CCLE database (https://portals.broadinstitute.org/ccle/about). (B) Western blot to detect the expression of GDH1, GS, and a-tubulin from 14 pairs of cancer and adjacent tissues of HCC. (C) Schematic diagram of cell model. 200, 000 HL7702 or 100, 000 HL-7702 and 100, 000 MHCC97H were cultured separately. (D) Relative urea excretion of the cell lines as indicated. (E) Relative ammonia abundance in the upper medium of the cell lines as indicated. (F) Relative ammonia abundance in the lower medium of the cell lines as indicated. (G) Relative urea abundance in 10 pairs of fresh HCC and adjacent tissues. (H) Relative ammonia abundance in 10 pairs of fresh HCC and adjacent tissues. [file Image_4.tif]

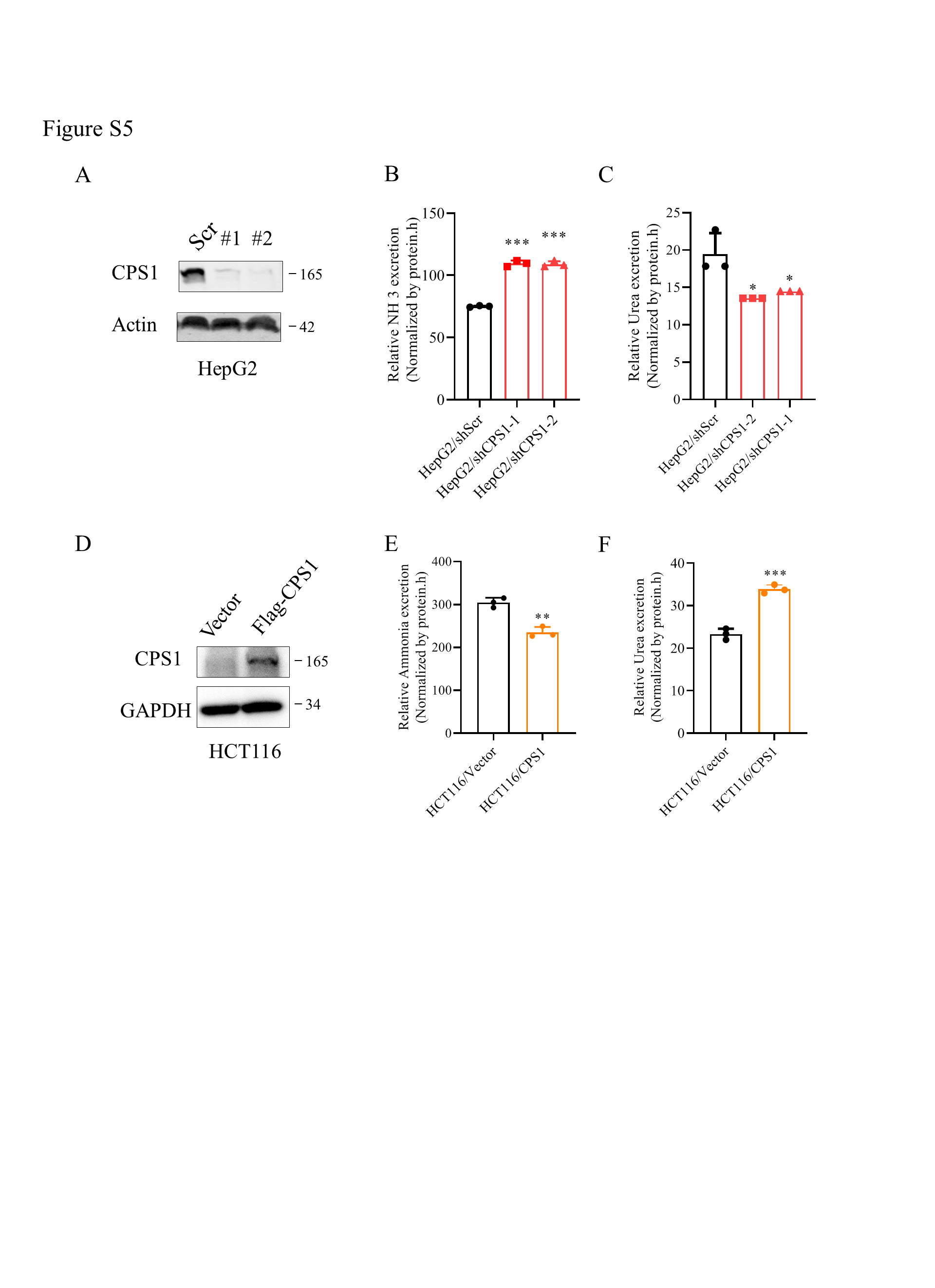

Supplement: Supplementary Figure 5 — The relationship of urea cycle with ammonia metabolism in cancer cells. (A) Western blot confirmed the knockdown of CPS1 in HepG2 cells. (B) Relative ammonia excretion in HepG2/Scramble, HepG2/shCPS1 for 8 h. (C) Relative urea excretion in HepG2/Scramble, HepG2/shCPS1 for 48 h. (D) Western blot confirmed the over-expression of CPS1 in HCT116 cells. (E) Relative ammonia excretion in HCT116/Vector, HCT116/CPS1 for 8 h. (F) Relative urea excretion in HCT116/Vector, HCT116/CPS1 for 48 h. Values are the means ± SD of three independent experiments. *p < 0.05, **p < 0.01, ***p < 0.001. [file Image_5.TIF]

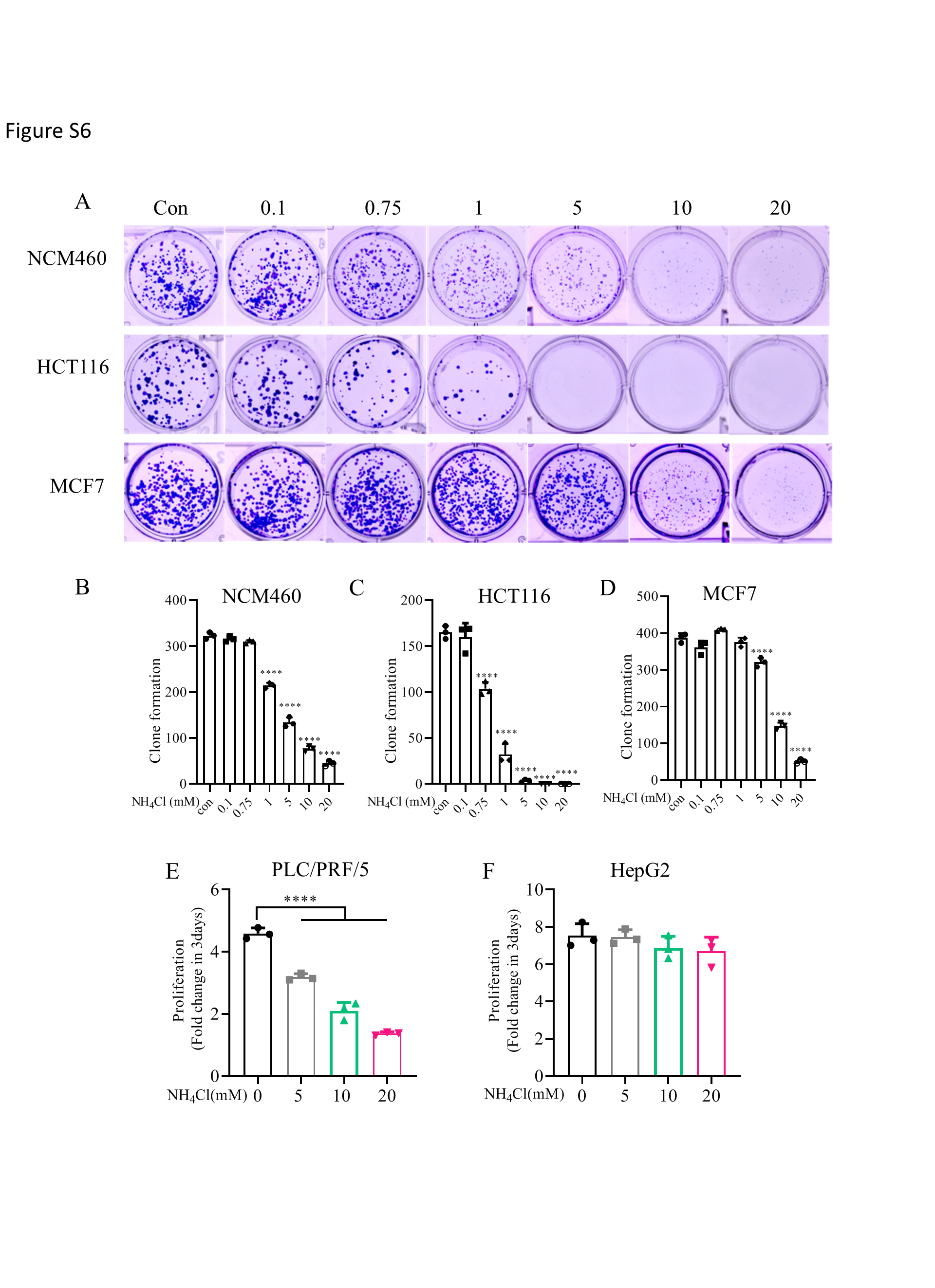

Supplement: Supplementary Figure 6 — High concentrations of ammonia inhibits the proliferation of cancer cells. (A) Colony formation ability of NCM460, HCT116 and MCF-7 under different concentrations of NH4Cl. (B–D) Colony number quantification of NCM460, HCT116, and MCF-7 under different concentrations of NH4Cl by Image J. (E,F) Proliferation of PLC/PRF/5 and HepG2 cells cultured under 0, 5, 10 or 20 mM NH4Cl for 3 days. Values are the means ± SD of three independent experiments. *p < 0.05; **p < 0.01, ***p < 0.001, ****p < 0.0001. [file Image_6.TIF]

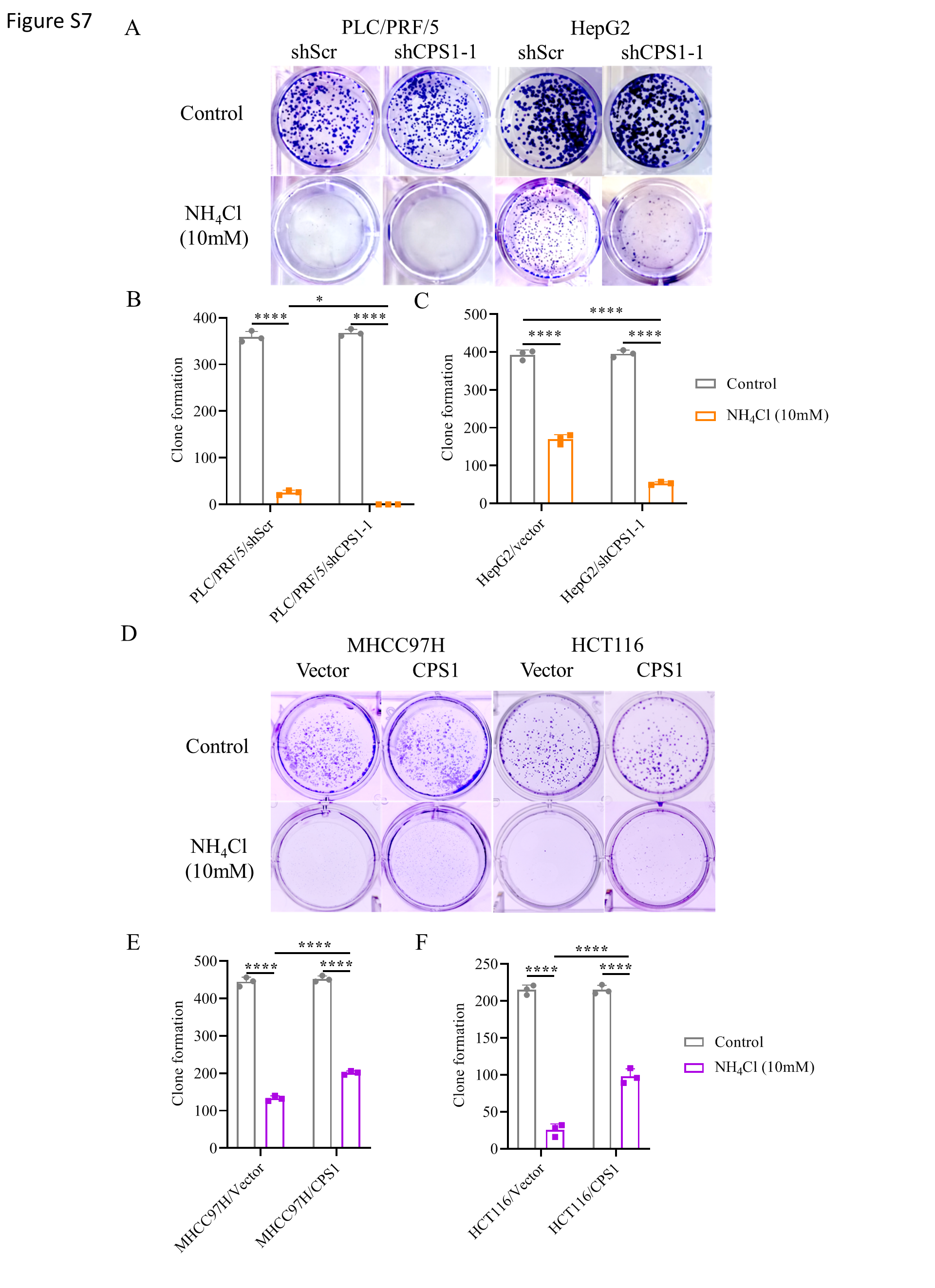

Supplement: Supplementary Figure 7 — Urea cycle protect cancer cells from high concentrations of ammonia. (A) Colony formation ability of PLC/PRF/5/shScr and PLC/PRF/5/shCPS1-1, HepG2/shScr and HepG2/shCPS1-1 under control or 10 mM NH4Cl. (B,C) Colony number quantification of PLC/PRF/5/shScr and PLC/PRF/5/shCPS1-1, HepG2/shScr and HepG2/shCPS1-1 under control or 10mM NH4Cl by Image J. (D) Colony formation ability of MHCC97H/Vector and MHCC97H/CPS1, HCT116/Vector and HCT116/CPS1 under control or 10mM NH4Cl. (E,F) Colony number quantification of MHCC97H/Vector and MHCC97H/CPS1, HCT116/Vector and HCT116/CPS1 under control or 10 mM NH4Cl by Image J. Values are the means ± SD of three independent experiments. *p < 0.05; **p < 0.01, ***p < 0.001, ****p < 0.0001. [file Image_7.TIF]
