# Supplementary material for: Urea as a By-Product of Ammonia Metabolism Can Be a Potential Serum Biomarker of Hepatocellular Carcinoma
Source: Front Cell Dev Biol. 2021 Apr 1;9:650748. doi: 10.3389/fcell.2021.650748 (PMC8047217; doi:10.3389/fcell.2021.650748)
Supplement: Supplementary Table 1 — Characteristics of serum samples from healthy controls, HBV, HBV positive cirrhosis, HCV, HBV positive HCC patients, and metastatic HCC patients. [file Data_Sheet_1.doc]

**Supplementary Table 1** Characteristics of serum samples from HCC, metastatic HCC patients and healthy controls

| Cancer type | Cases | Age | Sex(male/female) |
| --- | --- | --- | --- |
| Control | 115 | 59.62(53.00, 66.00) | 85/30 |
| HBV | 69 | 58.70(55.00, 61.50) | 51/18 |
| Cirrhosis | 108 | 58.38(54.25, 62.75) | 80/28 |
| HCV | 95 | 58.54(53.00, 64.00) | 70/25 |
| HCC | 294 | 59.36(53.00, 67.00) | 228/66 |
| Metastatic HCC† | 77 | 58.13(51.00, 64.50) | 57/20 |
| *P* |  | 0.7193 | 0.937 |

† Metastatic HCC refers other cancer metastasize to the liver.

**Supplementary Table 2** Characteristics of serum samples from lung cancer, breast cancer, colorectal cancer, HCC patients and healthy controls

| Cancer type | Cases | Age | Sex(male/female) |
| --- | --- | --- | --- |
| Control | 118 | 56.61(52, 62) | 78/40 |
| Lung cancer | 142 | 58.42(54, 63) | 88/54 |
| Breast cancer | 150 | 57.81(53, 62) | 0/150† |
| Colorectal cancer | 140 | 58.58(52, 66) | 92/48 |
| HCC | 165 | 57.05(51, 63) | 104/61 |
| *P* |  | 0.2324 | 0.754 |

†Sex difference analysis does not include breast cancer.
